# 17β-Estradiol Enhances Signalling Mediated by VEGF-A-Delta-Like Ligand 4-Notch1 Axis in Human Endothelial Cells

**DOI:** 10.1371/journal.pone.0071440

**Published:** 2013-08-13

**Authors:** Cristiana Caliceti, Giorgio Aquila, Micaela Pannella, Marco Bruno Morelli, Cinzia Fortini, Paolo Pinton, Massimo Bonora, Silvana Hrelia, Antonio Pannuti, Lucio Miele, Paola Rizzo, Roberto Ferrari

**Affiliations:** 1 Department of Medical Sciences, Cardiovascular Section, Azienda Ospedaliero-Universitaria, Arcispedale Sant’Anna, Laboratory for Technologies of Advanced Therapies (LTTA), University of Ferrara, Ferrara, Italy; 2 Department of Surgeon, Morphology and Experimental Medicine, Section of General Pathology, Interdisciplinary Center for the Study of Inflammation (ICSI), Laboratory for Technologies of Advanced Therapies (LTTA), University of Ferrara, Ferrara, Italy; 3 Department for Life Quality Studies, Alma Mater Studiorum - University of Bologna, Bologna, Italy; 4 University of Mississippi, Medical Center Cancer Institute, Jackson, Mississippi, United States of America; University of Valencia, Spain

## Abstract

Estrogens play a protective role in coronary artery disease. The mechanisms of action are still poorly understood, although a role for estrogens in stimulation of angiogenesis has been suggested. In several cell types, estrogens modulate the Notch pathway, which is involved in controlling angiogenesis downstream of vascular endothelial growth factor A (VEGF-A). The goal of our study was to establish whether estrogens modulate Notch activity in endothelial cells and the possible consequences on angiogenesis. Human umbilical vein endothelial cells (HUVECs) were treated with 17β-estradiol (E2) and the effects on Notch signalling were evaluated. E2 increased Notch1 processing as indicated by i) decreased levels of Notch1 transmembrane subunit ii) increased amount of Notch1 in nuclei iii) unaffected level of mRNA. Similarly, E2 increased the levels of the active form of Notch4 without altering Notch4 mRNA. Conversely, protein and mRNA levels of Notch2 were both reduced suggesting transcriptional repression of Notch2 by E2. Under conditions where Notch was activated by upregulation of Delta-like ligand 4 (Dll4) following VEGF-A treatment, E2 caused a further increase of the active form of Notch1, of the number of cells with nuclear Notch1 and of Hey2 mRNA. Estrogen receptor antagonist ICI 182.780 antagonized these effects suggesting that E2 modulation of Notch1 is mediated by estrogen receptors. E2 treatment abolished the increase in endothelial cells sprouting caused by Notch inhibition in a tube formation assay on 3D Matrigel and in mouse aortic ring explants. In conclusion, E2 affects several Notch pathway components in HUVECs, leading to an activation of the VEGF-A-Dll4-Notch1 axis and to a modulation of vascular branching when Notch signalling is inhibited. These results contribute to our understanding of the molecular mechanisms of cardiovascular protection exerted by estrogens by uncovering a novel role of E2 in the Notch signalling-mediated modulation of angiogenesis.

## Introduction

The Notch pathway is highly conserved from invertebrates to mammals [1] since it plays a crucial role in determining cell fate and differentiation during development and postnatal life. Mammals express four highly homologous receptors (Notch1, 2, 3 and 4) and five ligands (Delta-like ligand 1, 3, 4 and Jagged1, 2). Notch receptors are synthesized as single chain precursors that undergo a first proteolytic cut in the Golgi apparatus by a furin-like protease, after which mature heterodimeric receptors are transported to the cell membrane. The binding of a Notch ligand on the adjacent cell dissociates the extracellular subunit from the transmembrane subunit NotchTM. This allows the second proteolytic cut of NotchTM by a surface protease, generally ADAM10 (A Disintegrin And Metalloprotease 10), which creates a membrane-tethered intermediate (NEXT or Notch Extracellular Truncation) that is a substrate for the γ-secretase complex, an intramembranous aspartyl-protease complex. γ-secretase generates the active form of Notch (Notch intracellular, NotchIC), which translocates to the nucleus where it binds transcription factor CSL (CBF-1, Suppressor of Hairless, Lag-1), also known as RBP-Jκ (Recombinant Signal Binding Protein 1 for Jκ). NotchIC binding displaces a co-repressor complex, promotes the recruitment of co-activator molecules and activates transcription of Notch target genes such as Hes (hairy/enhancer of split), Hey (Hes-related proteins) and Nrarp (Notch-regulated ankyrin repeat protein). These factors, in turn, regulate downstream genes, some of which can either maintain cells in an uncommitted state or induce differentiation. Genes that control cell proliferation and apoptosis are also regulated by Notch activity [2].

Notch receptors 1, 2, 4 and ligands Delta-like 1 (Dll1), 4 (Dll4), Jagged1 are expressed in the endothelium and play a major role in the development and homeostasis of the vascular system [3]–[5]. The Notch pathway modulates vasculogenesis and neo-angiogenesis by cross-talks with the vascular endothelial growth factor receptors (VEGF-R). Under hypoxia, VEGF-A through VEGF-R2 induces filopodia formation on endothelial cells, leading to sprouting of new blood vessels from pre-existing ones. VEGF-A also induces Dll4 expression in the endothelium which, by activating Notch on adjacent cells, reduces expression of VEGF-R2 and limits sprouting. Notch signalling mediated by Jagged1 promotes instead sprouting. Depending on the ratio Dll4/Jagged1, Notch signalling will have different effects on angiogenesis [4]. Notch-dependent VEGF-R3 upregulation allows angiogenesis without VEGF-A-VEGF-R2 signalling [6]. Activation of Notch1 signalling is also involved in the protective effects of VEGF-A on endothelial cells, promoting survival under hypoxic conditions [7].

The endothelium is a major target for estrogens. In women, the onset of menopause coincides with increased risk of coronary artery disease, suggesting a protective effect of estrogens on vascular endothelium [8]. A large amount of pre-clinical data show that estrogen-mediated vascular protection is due at least in part to reduced endothelial cell dysfunction, promotion of endothelial healing and angiogenesis (reviewed in [9]). Altough it is known that the action of estrogens in the endothelium is mainly mediated by estrogen receptors (ERs), the downstream pathways involved in these protective effects are still incompletely understood.

In this study we sought to determine whether modulation of Notch signalling by estrogens could play a role in the protective effects of estrogens on the endothelium. Cross-talks between Notch and estrogen receptors have been shown in hormone responsive breast cancer cells [10], in the endometrium [11], [12], in hippocampal neurons [13] and in the dentate gyrus [14]. Modulation of Notch signalling in human umbilical vein endothelial cells (HUVECs) by 17β-estradiol (E2) has been previously reported [15], [16] but the details of this regulation have not been defined. We characterized by Western blotting, immunofluorescence staining and quantitative RT-PCR the effects of E2 treatment on each component of the Notch pathway expressed in HUVECs. We find that i) E2 affects Notch1, 2, 4 and Jagged1 protein levels and ii) specifically enhances Dll4-activated Notch1signalling. Moreover in HUVECs cultured on 3D Matrigel and in aortic ring explants, E2 counteracts increased endothelial sprouting caused by Notch inhibition and acts as a modulator of the sprouting angiogenesis driven by the VEGF-A-Dll4-Notch1 axis.

## Materials and Methods

### Materials

Antibodies to Notch1 (C-20), Notch4 (H-225), Jagged1 (H-114), VE-cadherin (F-8) and Estrogen Receptor α(MC-20) were from Santa Cruz Biotechnology (Santa Cruz, CA, USA). Antibodies to Dll1 (ab 84620) and Dll4 (ab7280) were from Abcam (Cambridge, UK). Antibodies to estrogen receptor β(5513), cleaved Notch1, valine 1744 (4147) were from Cell Signaling Technology (Beverly, MA, USA). Notch2 antibody (clone C651.6DbHN) was purchased from Developmental Studies Hybridoma Bank, University of Iowa (Iowa City, IA, USA). β-actin antibody was from Sigma Aldrich (St. Louis, MS, USA). Oligofectamine, Opti-MEMI reduced-serum medium, M200 medium, Low Serum Growth Supplements, ProLong Gold antifade reagent with DAPI, VEGF-A, Running and Transfer Buffers, ECL Plus Western Blotting Detection Reagents, SuperScript III reverse transcriptase, Random Hexamers, dNTPs, RNase Out were from Life Technologies (Carlsbad, CA, USA). EGM-2 bullet-kit and Fetal Bovine Serum (FBS) were from Lonza (Basel, Switzerland). RNeasy Mini Kit was from Qiagen (Hilden, Germany). PerfeCta SYBR Green SuperMix with ROX kit were from Quanta Biosciences (Gaithersburg, MD, US). Primers for RT-PCR were purchased from IDT (Coralville, IA, USA). Other materials were purchased from Sigma Aldrich. All the other chemicals and solvents were of the highest analytical grade.

### Cell Culture

HUVECs pools, purchased from Life Technologies, were plated on gelatin-coated tissue culture dishes and maintained in phenol red-free basal medium M200 (Life Technologies) containing 10% FBS and growth factors (LSGS, Life Technologies) at 37°C with 5% CO_2_. Cells from passages 3 to 7 were actively proliferating (70–90% confluent) when samples were harvested and analyzed. HUVECs were hormone-deprived using charcoal/dextran-treated FBS (csFBS) for 16 hours before 24 hours treatment with 17β-estradiol (E2). We used 3 different conditions to hormone-deprive and treat cells: M1 (10% csFBS), M2 (20% csFBS), M3 (10% csFBS and growth factors, LSGS, Life Technologies). As an alternate approach, HUVECs were synchronized in medium supplemented with 2% FBS for 16 hours followed by E2 treatment for 24 hours in medium supplemented with 20% csFBS (M4). Lastly, we conducted experiments using VEGF-A- containing medium. Briefly, cells were hormone-deprived for 16 hours and then treated with E2 for 24 hours in medium supplemented with 2% csFBS and growth factors (EGM-2, Lonza) (M5).

10^−9 ^M 17β-estradiol (Sigma Aldrich) solubilized in DMSO was used. Notch activity was inhibited by treatment with γ-secretase complex inhibitor DAPT (LY-374973, Sigma Aldrich). DAPT was dissolved in DMSO to a stock concentration of 5 mM and diluted to a final concentration of 5 µM in culture medium just prior to use. 10^−7 ^M ERs antagonist ICI 182.780 (fulvestrant, Sigma Aldrich) solubilized in DMSO was used to evaluate whether the observed effects were mediated by ERs.

### Western Blotting and Densitometric Analysis

Western blot analyses were carried out to detect expression of Notch1, Notch4, Notch2, Jagged1, Dll1, Dll4, ERα, ERβ and β-actin by using the corresponding antibodies. Cells were lysed in RIPA Buffer (0.05% sodium-deoxycholate was freshly added) containing 10 µg/ml aprotinin, 10 µg/ml leupeptin, 10 µg/ml pepstatin A, 1 mM PMSF and 1 mM sodium orthovanadate on ice for 30 minutes. Protein samples were denatured by incubation at 70°C for 10 minutes in sample buffer (Life Technologies) containing 0.5 M DTT adding and separated on 7.5% NuPAGE gels (Life Technologies). Proteins were transferred to PVDF membrane at 30 V for 150 min. Non-specific binding was blocked by incubating membranes with Tris-buffered saline (TBS)/Tween, pH 8.0, containing 5% nonfat dry milk for 1 hour at room temperature. PVDF membranes were incubated overnight at 4°C with primary antibodies, washed 3 times in TBS/Tween, and incubated for 60 minutes at room temperature with secondary antibodies peroxidase-conjugated in TBS/Tween containing 5% nonfat dry milk. Membranes were washed and developed using ECL Plus Western Blotting Detection Reagents (Life Technologies). Images of the blots were obtained exposing them to X-ray film (Kodak, Sigma Aldrich). MCF-7 cells lysate was purchased from Cell Signaling Technology. Protein immunoreactive bands were analyzed and quantitated by using the Image J analysis software. Values were expressed as the ratio of each band density to control band density normalized to control.

### RNA Extraction

HUVECs, treated for 16–24 hours with phenol red-free M200 medium containing csFBS, were exposed to vehicle or 17β-estradiol in phenol red-free M200 medium containing csFBS for 24 hours. Total RNA was extracted using a commercially available kit (Qiagen).

### Real-Time PCR

500 ng of total RNA were reverse transcribed in a volume of 25 µl using 250 units of SuperScript III reverse transcriptase and 50 ng of random hexamers. Reaction conditions were as suggested by manufacturer. 2 µl of the cDNA mixture were used for real-time PCR experiments to measure the amount of Notch1, Notch2, Notch4, Hes1, Hes4, Hey1, Hey2, HeyL, Jagged1 and Dll4 transcripts. Real-time PCR reactions were conducted on an Applied Biosystems 7500 Fast Real-Time PCR System using PerfeCta SYBR Green SuperMix with ROX kit (Quanta Biosciences) according to the manufacturer’s protocol in a final volume of 25 µl. Primers concentration was 500 nM. The following primers were used: Notch1: forward 5′-GTCAACGCCGTAGATGACC-3′, reverse 5′- TTGTTAGCCCCGTTCTTCAG-3′, Notch2: forward 5′-CAGATGCGAGTGTGTCCCAGGCT-3′, reverse 5′-TACCCCGAGTGCCTGGTGGGC-3′; Notch4: forward 5′-CAACTGCCTCTGTCCTGATG-3′, reverse 5′-GCTCTGCCTCACACTCTG-3′; Hes1: forward 5′-CGGACATTCTGGAAATGACA-3′, reverse 5′-CATTGATCTGGGTCATGCAG-3′; Hes4: forward 5′-CCTCAGAAAAGAGAGCTCCCGCC-3′, reverse 5′-GGCGCGGTACTTGCCCAGAA-3′; Hey1: forward 5′-TGAGCTGAGAAGGCTGGTAC-3′ reverse 5′-ATCCCAAACTCCGATAGTCC-3′: Hey2: forward 5′-AAAAGGCGTCGGGATCG-3′, reverse 5′-AGCTTTTTCTAACTTTGCAGATCC-3′; HeyL: forward 5′-AAGAGGGCCAGCTGAGCCAGA-3′, reverse 5′-GATGCGGTCTCGACGCCGTT-3′; Jagged1: forward 5′-GACTCATCAGCCGTGTCTCA-3′, reverse 5′-TGGGGAACACTCACACTCAA-3′; Dll4: forward 5′-CTGTGCCAACGGGGGACAGTG-3″, reverse 5′-GTGGGCGCAAGGGTTACGGG-3′;. RPL13A forward 5′-GGAGGTGCAGGTCCTGGTGCTT-3′, reverse 5′-CGTACGACCACCACCTTCCGG-3′. Changes in gene expression were calculated by the 2^−ΔΔCt^ formula using RPL13A as reference gene.

### Short Interfering (si)RNA Transfection

HUVECs were grown to 40% confluence in 100-mm dishes and transfected with 10 nM of scrambled control (si)RNA (Santa Cruz Biotechnologies) or Notch4 (si)RNA (Santa Cruz Biotechnologies) using Oligofectamine (Life Technologies), as previously described [17]. Cells were used for experiments at 48 hours after transfection.

### Cell Viability

Viable cells were evaluated by the MTT assay, since the reduction of tetrazolium salts is widely accepted as a reliable way to examine cell viability/proliferation [18]. Cells (2×10^4^) were incubated in 96-well flat-bottomed plates with 0.5 mg/ml MTT for 4 hours at 37°C. At the end of the incubation, blue-violet formazan salt crystals were formed and dissolved by adding solubilization solution (10% SDS, 0.01 M HCl). Plates were then incubated overnight in humidified atmosphere (37°C, 5% CO_2_) to ensure complete lysis. The absorbance at 570 nm was measured using a multiwell plate reader (Elisa plate reader, Thermo Scientific).

### Immunofluorescence

HUVECs grown on glass slides were rinsed in ice cold PBS, fixed in acetone for 10 min, rinsed twice in PBS and then blocked in 3% BSA/PBS for 1hour at room temperature. Cells were then incubated with the relative antibodies overnight at 4°C, rinsed twice in PBS followed by 1 hour treatment with 488-conjugated goat anti-rabbit IgG (Alexa Fluor-Life Technologies) and 596-conjugated rabbit anti-mouse IgG (Alexa Fluor-Life Technologies) at room temperature. After washing in PBS, ProLong Gold antifade reagent containing DAPI (Life Technologies) mounting medium was added. Images were acquired on Olympus Scan^R station, using a laser based hardware and an image-based automatic autofocus. 50 fields were acquired for each well using a 40X magnification (N.A. 0.95). Different fluorophores were excited using 377/50, 498/20, 595/30 bandpass filters for DAPI, FITC and TRITC respectively. Images were collected using an Orca-05G2 at full-frame, without binning. Cells were then scored and counted using the Scan^R Analysis software.

### HUVECs Tube Formation Assay

HUVECs (passage 4–6) grown to 70–80% confluency in phenol red- free M200 containing 2% FBS and EGM-2 were utilized. Cells were hormone-deprived for 8 hours in phenol red-free M200 containing 2% csFBS/EGM-2 and treated overnight with 1 nM E2, 5 µM DAPT, 1 nM E2 plus 5 µM DAPT or with DMSO. Cells were then trypsinized and 9×10^4^ cells were resuspended in fresh media containing their respective treatments and added to wells of a 24 wells plate containing 400 µL of reduced growth factor Matrigel™ matrix (B&D Biosciences, Bedford, MA, USA). The plates were incubated at 37°C and tube formation was monitored. After 8 hours of tube formation, images were captured with a Nikon Digital Sight DS-2Mv camera coupled to a light inverted microscope (4X objective). Endothelial cells sprouting was assessed by counting the number of closed intercellular compartments (closed rings or pro-angiogenic structures) in 4 fields per wells, as previously described [19].

### Mouse Aortic Ring Assay

*Ex vivo* sprouting angiogenesis was studied by culturing 1-mm rings of mouse aorta in rat type I collagen (Sigma) as described [20]. These experiments were approved by the University of Ferrara Ethic Committee for Animal Research. Thoracic aortas were removed from euthanized C57BL/6 mice, flushed gently with 500 µl of Opti-MEM/GlutaMAX-I medium (Life Technologies) and gently stripped of periaortic fibroadipose tissue. Aortas were sectioned into 1-mm length rings (∼15 per aorta), which were embedded in 1 mg/ml of rat tail type I collagen gel (Sigma) in 1X DMEM containing 100 units/ml penicillin and 100 ng/ml streptomycin. Gels containing the aortic rings were polymerized in a 96-well plate by incubation at 37°C for 1 hour. 150 µl of Opti-MEM/GlutaMAX-I medium supplemented with 2.5% csFBS and VEGF-A (30 ng/ml) and the specific treatment (DMSO, 1 nM E2, 5 µM DAPT and 1 nM E2 plus 5 µM DAPT) was added to the collagen. Each treatment was performed in triplicate. Aortic rings were incubated at 37°C in a humidified incubator for up to 1 week, with media changes every other day. Vascular sprouting from each ring was examined daily on a Nikon Eclipse TE300 microscope (4X objective). Quantitative analysis of endothelial sprouting was performed at day 5 and 7 by measuring sprout length using a calibrated micrometer with the Nikon NIS-elements D3.1 software. The greatest distance from the aortic ring body to the end of the vascular sprouts was measured at three distinct points per ring and in three different rings per treatment, as previously described [21].

### Statistical Analysis

Results are expressed as mean ± SEM of at least three independent experiments. Differences between the mean were determined by two-tailed Student's t test and a P value <0.05 was considered to be statistically significant.

## Results

### Expression of ERα and ERβ in HUVECs

**Figure 1:**
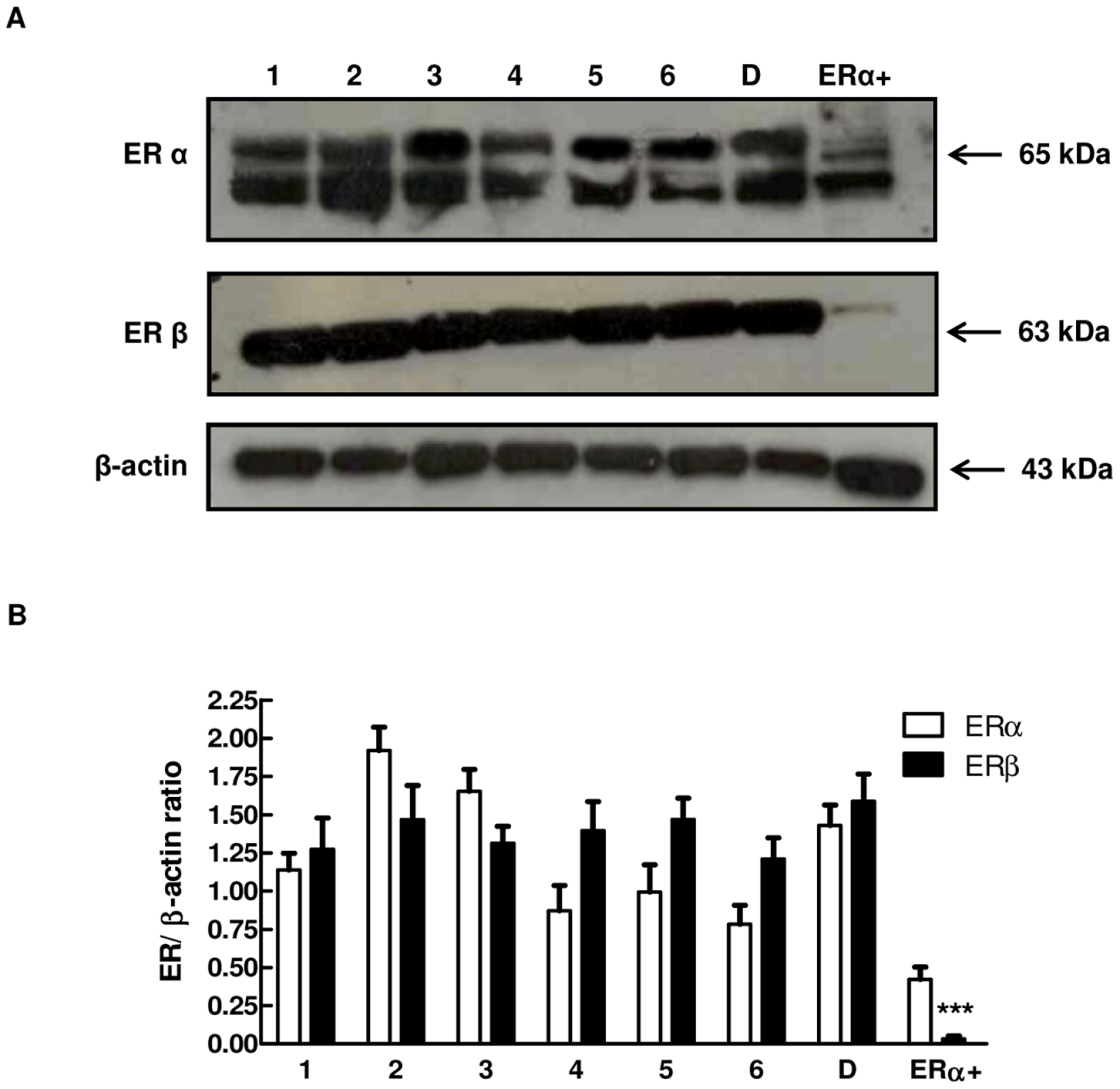
Expression of Estrogen Receptors in HUVECs. (A) Cell lysates obtained from different pools of commercially available HUVECs (1–6) and HUVECs obtained from a single donor (D) were electrophoresed and immunoblotted with ERα and ERβ antibodies. β-actin antibody was used to ensure equal loading. ERα^+^: estrogen receptor α positive cells (MCF-7 cells). (B) Densitometric analysis of Western blot assay to quantify ERα and ERβ protein levels. Results are expressed as mean ± SEM of three independent experiments, ***P<0.001 significantly different from the ERα levels in MCF7 cells.

It has been reported in other cell types that regulation of Notch signalling by 17β-estradiol is mediated by estrogen receptors [10], [14]. Previous reports on the ER status of HUVECs have been contradictory; some authors have shown expression of both receptors [22] where others have shown expression of only ERβ [23], [24]. Therefore, we evaluated by Western blot analysis the expression of ERα and ERβ in 6 different pools of commercially available HUVECs and in cells derived from the umbilical vein of a single donor. Figure 1 shows that both ERs are expressed at similar levels in every batch we analyzed. The antibody against ERβ detected a single band (63 kDa) while the antibody against the COOH-terminus of the ERα detected two major bands at approximately 65 and 50 kDa, as previously described in HUVECs by Russell and colleagues [25]. Unlike MCF-7 breast cancer cells, which express negligible levels of ERβ, the HUVECs utilized in our study express comparable amount of ERα and β.

### Effect of 17β-estradiol Treatment on the Components of Notch Pathway Expressed in HUVECs

**Figure 2:**
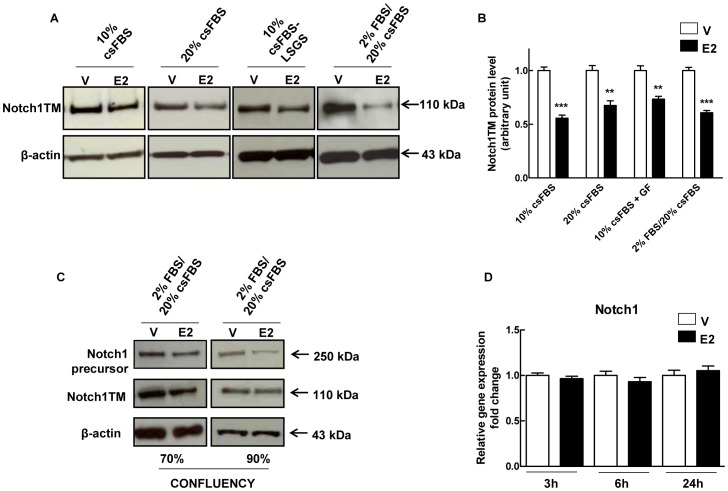
Effect of 17β-estradiol treatment on Notch1 processing in HUVECs under different experimental conditions. (A) HUVECs were treated with 1 nM 17β-estradiol (E2) or DMSO (V) for 24 hours under different experimental conditions as described in the Materials and Methods section. Cell lysates were electrophoresed and immunoblotted with Notch1 (C-20) antibody to detect changes in the transmembrane form of Notch1 (Notch1TM). β-actin antibody was used to ensure equal loading. (B) Densitometric analysis of Western blot assay to quantify Notch1TM protein levels. Results are expressed as mean ± SEM of three independent experiments, **P<0.01, ***P<0.001 significantly different from the control. (C) HUVECs at different confluence (70% vs. 90%) were treated with 1 nM E2 or DMSO (V) for 24 hours under M4 experimental conditions (2% FBS overnight followed by 20% csFBS). Cell lysates were electrophoresed and immunoblotted with Notch1 (C-20) antibody to detect changes in the precursor (Notch1 precursor) and the transmembrane (Notch1TM) form. β-actin antibody was used to ensure equal loading. Densitometric analysis of Western blot assay is shown in Figure S6A. (D) HUVECs were treated with 1 nM E2 or DMSO (V) for 3, 6 and 24 hours under M4 experimental conditions (2% FBS overnight followed by 20% csFBS). Total RNA was extracted and qRT-PCR analysis of Notch1 gene expression was performed. Relative changes in mRNA expression levels were calculated according to the 2^−ΔΔCt^ method using RPL13A as reference gene. Results are expressed as mean ± SEM of three independent experiments, each performed in triplicate.

A variety of experimental conditions have been used to study the actions of estrogens in HUVECs [15], [16], [26]–[28]. Since hormone deprivation commonly used before E2 treatment strongly reduced cell viability, we compared different conditions in order to achieve maximum response to E2 without affecting cell viability (see Materials and Methods for the different conditions utilized). Western blot analysis utilizing an antibody directed against the C-terminus of the Notch1 protein, showed that under every condition tested, E2 treatment markedly decreased the intensity of a ∼110 kDa band which represents the transmembrane form of Notch1 (Notch1TM) receptor (Figure 2A). Assessment of cell viability by MTT (data not shown) showed that M4 conditions (2% FBS for 16 hours followed by E2 treatment for 24 hours in medium supplemented with 20% csFBS) were optimal, since they induced the strongest Notch1TM decrease (∼40%) (Figure 2B) while cell viability was nearly unaffected. These conditions were chosen for subsequent experiments. E2 treatment reduced the Notch1 precursor protein and Notch1TM in cells grown to 70% (∼38% and ∼43% reduction, respectively) and 90% confluency (∼67% and ∼19% respectively) (Figures 2C and S6A) suggesting that the observed effect on Notch1 was not an artifact related to cell density [29]. The antibody specific for Notch1 cleaved at valine 1744, the active form of Notch1 (Notch1IC), didn’t detect any bands, suggesting low steady-state levels of Notch1IC under M4 conditions, or an alternate γ-secretase cleavage site (Figure S1). Notch1 mRNA levels measured by qRT-PCR were not affected after 3, 6 and 24 hours of E2 treatment (Figure 2D), indicating that E2 induces post-translational modulation of Notch1.

**Figure 3:**
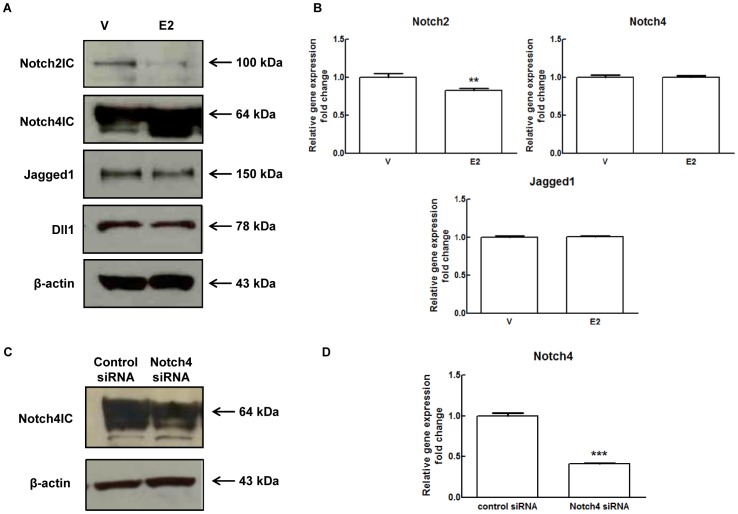
Effect of 17β-estradiol treatment on Notch pathway components in HUVECs. (A) HUVECs were treated with 1 nM E2 or DMSO (V) for 24 hours under M4 experimental conditions (2% FBS overnight followed by 20% csFBS). Cell lysates were electrophoresed and immunoblotted with antibodies for Notch2, Notch4, Jagged1, Dll1. β-actin antibody was used to ensure equal loading. Densitometric analysis of Western blot assay is shown in Figure S6B. (B) HUVECs were treated with 1 nM E2 or DMSO (V) for 24 hours under M4 experimental conditions (2% FBS overnight followed by 20% csFBS). Total RNA was extracted and qRT-PCR analysis of Notch2, 4 and Jagged1 genes expression was performed. Relative changes in mRNA expression levels were calculated according to the 2^−ΔΔCt^ method using RPL13A as reference gene. Results are expressed as mean ± SEM of three independent experiments, each performed in triplicate. **P<0.01, significantly different from the control. (C) Western blot analysis for Notch4 in HUVECs after Notch4 (si)RNA treatment for 48 hours. Lysates were immunoblotted with Notch4 antibody. β-actin antibody was used to ensure equal loading. Densitometric analysis of Western blot assay is shown in Figure S6C. (D) qRT-PCR analyses were performed to detect reduction of Notch4 mRNA levels in HUVECs after Notch4 (si)RNA treatment for 48 hours. Relative changes in mRNA expression levels were calculated according to the 2^−ΔΔCt^ method using RPL13A as reference gene. Results are expressed as mean ± SEM of three independent experiments, each performed in triplicate. ***P<0.001, significantly different from the control.

We then evaluated the effects of E2 on the other Notch receptors and ligands expressed in endothelium. In E2 treated cells, an antibody against the C-terminus of Notch4 protein detected an increase (∼53%) in a 64 kDa band and in a band immediately below (∼50 kDa) (Figures 3A and S6B). Whereas the 64 kDa band corresponds to the active form of Notch4 (Notch4IC) [30] no published data exist on the identity of the lower band. (si)RNA for Notch4 decreased the intensity of both bands (∼49%) confirming that they are both related to Notch4 (Figures 3C and S6C). As expected, Notch4 mRNA drastically decreased in cells treated with Notch4 (si)RNA compared to control (Figure 3D). qRT-PCR analysis showed that Notch4 mRNA was unchanged following E2 treatment (Figure 3B), suggesting that the increase in the two bands could be either due to stabilization of active Notch4 or to increased translation of the mRNA. E2 also decreased by ∼46% the intensity of the 100 kDa band corresponding to the active form of Notch2 (Notch2IC) [31] (Figures 3A and S6B) but, unlike the other two receptors, Notch2 mRNA levels were reduced by E2 (0.8-fold compared to control) (Figure 3B). Among ligands, Jagged1 processing was affected by E2, since we observed a reduction (∼23%) of the 150 kDa band corresponding to full length Jagged1 (Figures 3A and S6B) while Jagged1 mRNA levels were unchanged (Figure 3B). Under these experimental conditions, we could not detect the Dll4 70 kDa band corresponding to the mature form of Dll4 [32]. Lack of VEGF-A in our medium is consistent with our observation and in agreement with other reports showing undetectable expression of Dll4 in HUVECs grown in absence of VEGF-A [32]. Dll1 protein was not affected by E2, as shown in Figures 3A and S6B.

Taken together, these data show a generalized effect of E2 treatment on several components of the Notch pathway expressed in HUVECs.

### 17β-estradiol Increases the Activation of Notch1

Following E2 treatment, Western blots clearly showed an increase of active Notch4 and a reduction of active Notch2. As for Notch1, the reduction of Notch1 precursor and Notch1TM may be explained by a repression of Notch1 translation, increased processing toward the active form of the protein, or an increase of its degradation. In order to distinguish among these possibilities, we determined the levels of nuclear/cytoplasmic partitioning of Notch1 by immunofluorescence microscopy analysis. Immunofluorescence staining for Notch1 showed that in presence of E2 there was a higher number of cells with nuclei strongly positive for Notch1 compared to untreated cells (11.3±1.02 vs 7.3±0.53), in which Notch1 signal was predominantly localized to the plasma membrane (Figure S2A, B). Augmented Notch1 nuclear localization suggests that E2 increases processing toward the active form of Notch1 or stabilizes Notch1IC.

### 17β-estradiol Enhances Delta like Ligand 4-induced Notch Signalling in Endothelial Cells

**Figure 4:**
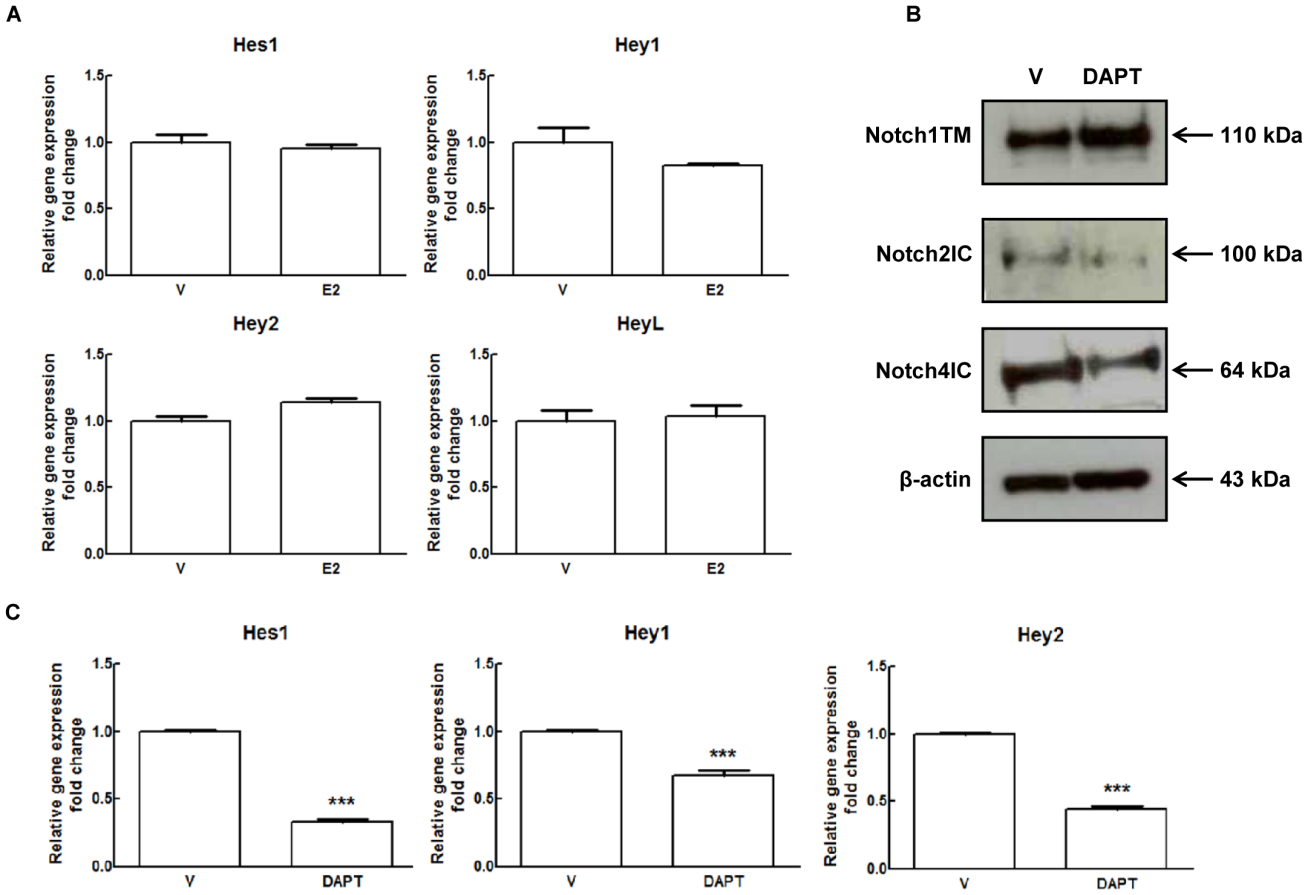
Effect of 17β-estradiol treatment on Notch canonical target genes in HUVECs. (A) HUVECs were treated with 1 nM E2 or DMSO (V) for 24 hours under M4 experimental conditions (2% FBS overnight followed by 20% csFBS). Total RNA was extracted and qRT-PCR analysis of Hes1, Hey1, Hey2 and HeyL genes expression was performed. Relative changes in mRNA expression levels were calculated according to the 2^−ΔΔCt^ method using RPL13A as reference gene. Results are expressed as mean ± SEM of three independent experiments, each performed in triplicate. (B) HUVECs were treated with 5 µM DAPT for 24 hours under M4 experimental conditions (2% FBS overnight followed by 20% csFBS). Cell lysates were electrophoresed and immunoblotted with Notch1 (C-20), Notch2 (clone C651.6DbHN) and Notch4 (H-225) antibodies to detect the transmembrane form of Notch1 (Notch1TM), the active form of Notch2 (Notch2IC) and the active form of Notch4 (Notch4IC). β-actin antibody was used to ensure equal loading. Densitometric analysis of Western blot assay is shown in Figure S6D. (C) HUVECs were treated with 5 µM DAPT for 24 hours under M4 experimental conditions (2% FBS overnight followed by 20% csFBS). Total RNA was extracted and qRT-PCR analysis of Hes1, Hey1 and Hey2 genes expression was performed. Relative changes in mRNA expression levels were calculated according to the 2^−ΔΔCt^ method using RPL13A as reference gene. Results are expressed as mean ± SEM of three independent experiments, each performed in triplicate. **P<0.01, significantly different from the control.

**Figure 5:**
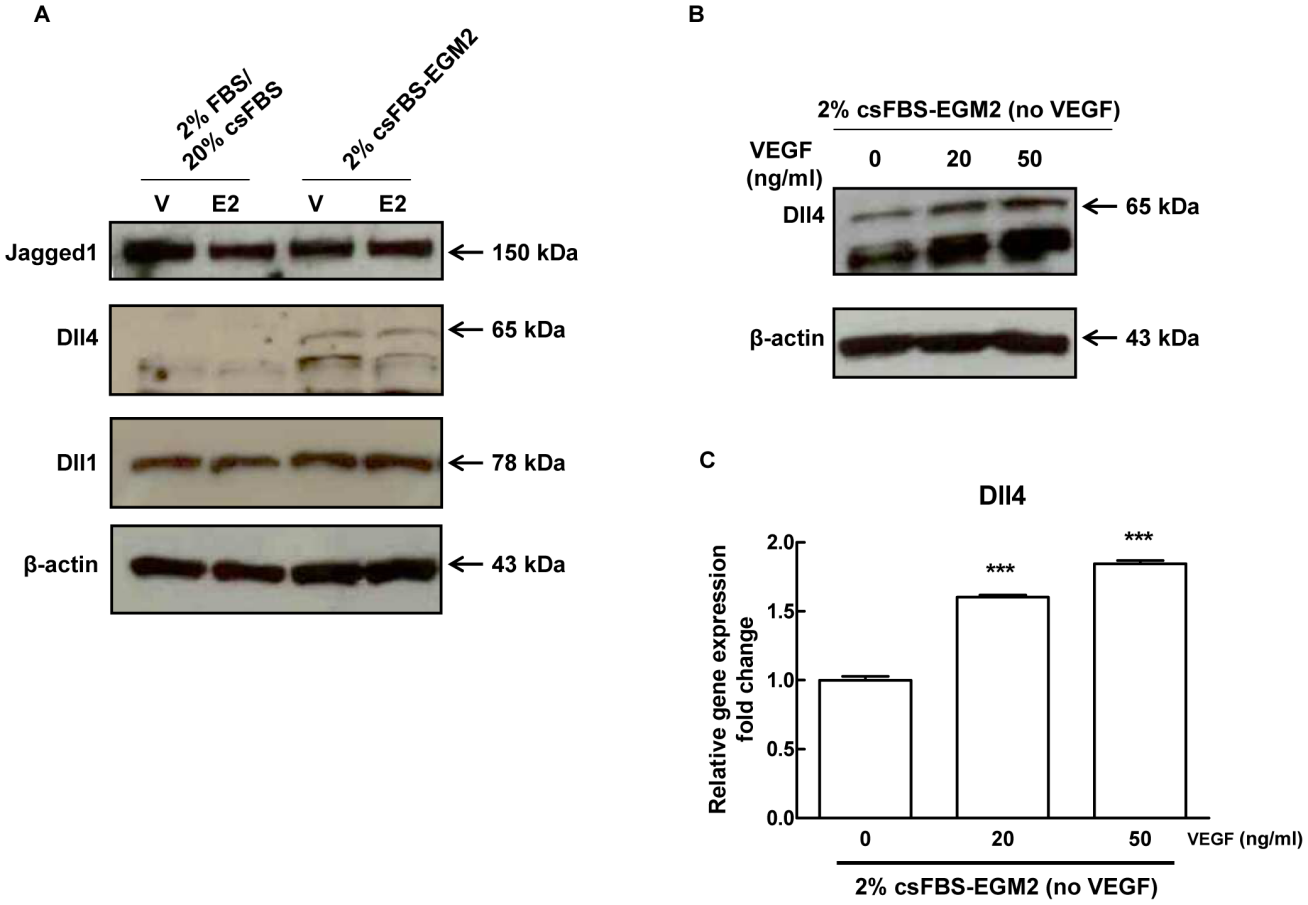
Effect of 17β-estradiol treatment on Notch ligands in HUVECs under different experimental conditions. (A) HUVECs were treated with 1 nM E2 or DMSO (V) for 24 hours under M4 (2% FBS overnight followed by 20% csFBS) or M5 (2% csFBS-EGM-2) experimental conditions. Cell lysates were electrophoresed and immunoblotted with Jagged1, Dll4 and Dll1 antibodies. β-actin antibody was used to ensure equal loading. Densitometric analysis of Western blot assay is shown in Figure S6E. (B) HUVECs were exposed to different VEGF-A concentrations (20 ng/ml and 50 ng/ml) for 24 hours under M5 experimental conditions (2% csFBS-EGM-2). Cell lysates were electrophoresed and immunoblotted with Dll4 antibody. β-actin antibody was used to ensure equal loading. Densitometric analysis of Western blot assay is shown in Figure S6F. (C) HUVECs were treated with VEGF-A (20 ng/ml and 50 ng/ml) for 24 hours under M5 experimental conditions. Total RNA was extracted and qRT-PCR analysis of Dll4 gene expression was performed. Relative changes in mRNA expression levels were calculated according to the 2^−ΔΔCt^ method using RPL13A as reference gene. Results are expressed as mean ± SEM of three independent experiments, each performed in triplicate. *** P<0.001, significantly different from control.

**Figure 6:**
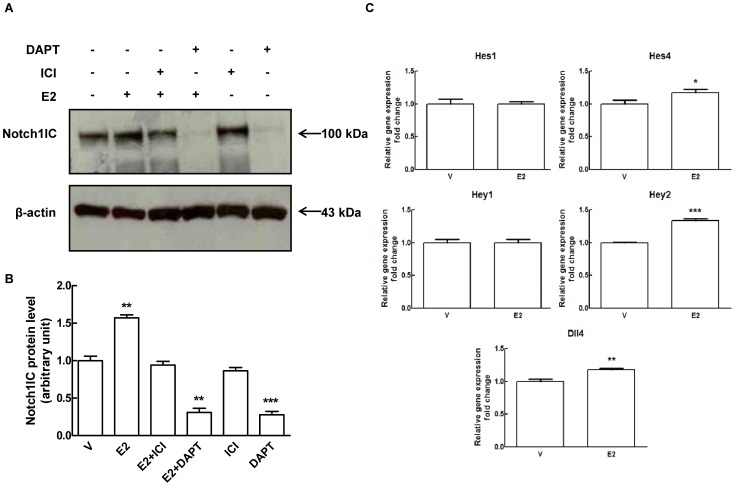
Effect of 17β-estradiol treatment on Notch1 processing in presence of Delta-like ligand 4 in HUVECs. (A) HUVECs were treated with 1 nM E2, 0.1 µM ICI 182.780, 5 µM DAPT, 1 nM E2 plus 0.1 µM ICI 182.780, 5 µM DAPT plus 1 nM E2 or DMSO (V) for 24 hours under M5 experimental conditions (2% csFBS-EGM-2). Cell lysates were electrophoresed and immunoblotted with cleaved Notch1 (Val1744) antibody. β-actin antibody was used to ensure equal loading. (B) Densitometric analysis of Western blot assay to quantify Notch1IC protein levels. Results are expressed as mean ± SEM of three independent experiments, **P<0.01, ***P<0.001 significantly different from the control. (C) HUVECs were treated with 1 nM E2 for 24 hours under M5 experimental conditions (2% csFBS-EGM-2). Total RNA was extracted and qRT-PCR analysis of Hes1, Hes4, Hey1, Hey2 and Dll4 genes expression was performed. Relative changes in mRNA expression levels were calculated according to the 2^−ΔΔCt^ method using RPL13A as reference gene. Results are expressed as mean ± SEM of three independent experiments, each performed in triplicate. * P<0.05; ** P<0.01; *** P<0.001, significantly different from control.

**Figure 7:**
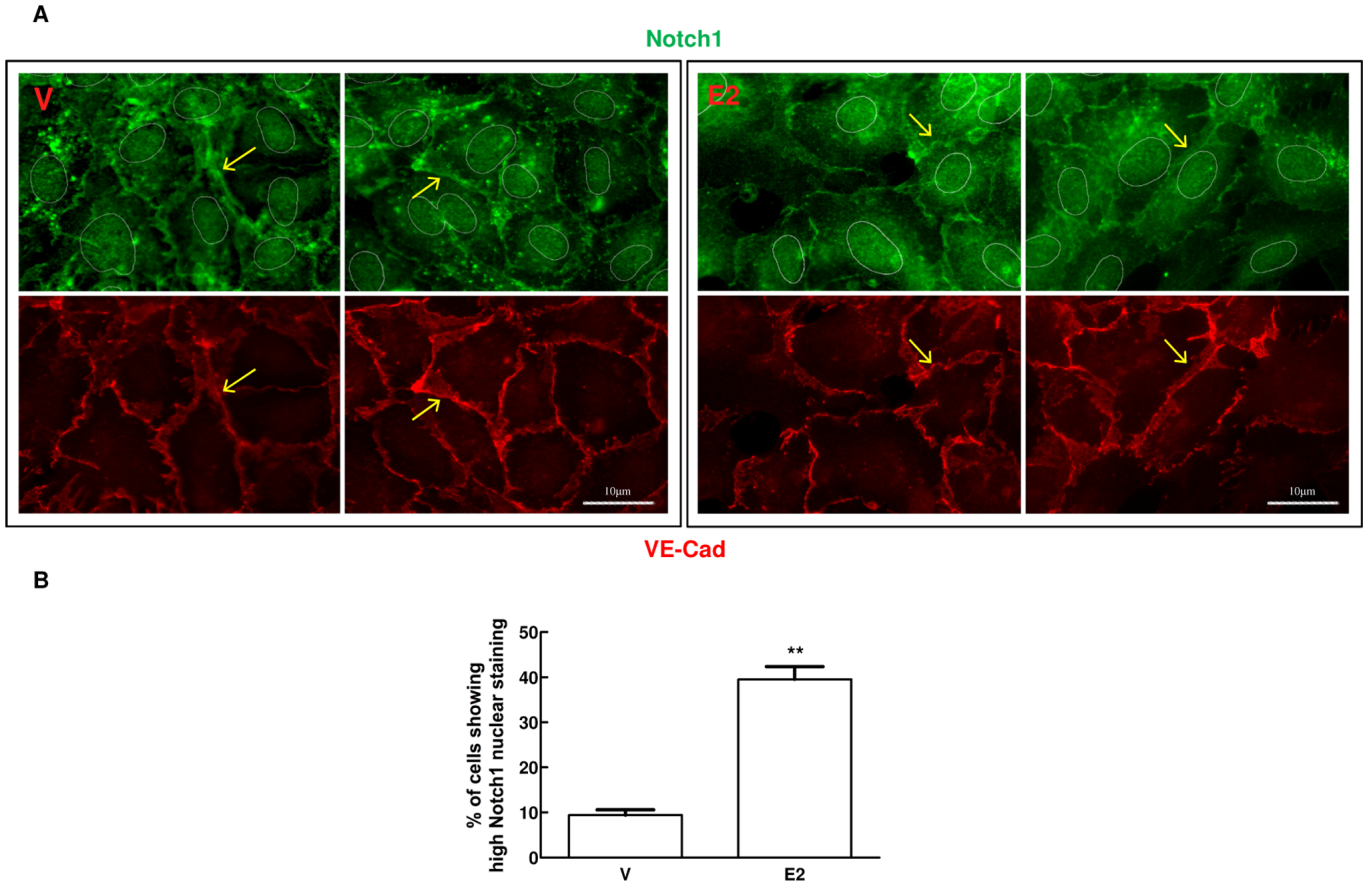
Effect of 17β-estradiol treatment on cellular Notch1 localization in HUVECs under M5 conditions. (A) Representative microscopy images of HUVECs immunolabelled with Notch1 (C-20) and VE-cadherin antibodies, then treated with 488-conjugated goat anti-rabbit IgG and 596-conjugated rabbit anti-mouse IgG secondary antibodies. DAPI staining was used to visualize margins of nuclei (indicated by dotted white lines). Yellow arrows point at Notch1 cytoplasmic membrane staining. Before immunofluorescence staining, cells were treated with 1 nM E2 or DMSO (V) for 24 hours under M5 experimental conditions (2% csFBS-EGM-2). (B) Percentage of cells showing high nuclear staining for Notch1. Cells were collected using an Orca-05G2 at full-frame, without binning. Cells were then scored and counted in 50 fields using the Scan^R Analysis software. Results are expressed as mean ± SEM of three independent experiments. **P<0.01, significantly different from the control.

Next we determined the effect of E2 on Notch transcriptional activity by measuring the expression levels of canonical Notch target genes Hes1, Hey1, Hey2 and HeyL. As shown in Figure 4A, in contrast with protein data shown in Figures 2A, C and 3A indicating an increase in Notch processing, we did not detect changes in Notch target genes other that a slight increase in Hey2 mRNA which didn’t reach statistical significance. Notch inhibition by DAPT (a γ-secretase complex inhibitor) (Figures 4B, C and S6D) confirmed that in our system Hes1, Hey1 and Hey2 were bona fide Notch targets. EDTA treatment, which causes ligand-independent Notch1 activation [33], strongly activated Notch1 as indicated by the appearance of the Notch1IC corresponding band (Figures S3A and S6G) and induced Hey1 and Hey2 mRNAs (4.7-fold and 2.9-fold respectively compared to control) (Figure S3B). Under these conditions, Hes1 mRNA was unchanged (Figure S3B). In the presence of E2, EDTA induced a stronger processing of Notch1 compared to EDTA-only control (∼71% increase of Notch1IC) (Figures S3C and S6H). As for target genes, the expression levels of Hey1 and Hes1 were unaffected, while Hey2 mRNA levels were further increased (1.5-fold) compared to EDTA-only control (Figure S3D). Even though EDTA treatments affects many pathways, these proof-of-concept experiments suggested that E2 might be able to increase Notch1 transcriptional activity downstream of ligand. The lack of transcriptional activation of Notch target genes by E2 in absence of EDTA treatment could then be due to the fact that Dll4 was undetectable under these conditions. Therefore we compared the expression of Notch ligands under the conditions used up to that point (M4) and in HUVECs grown in medium containing VEGF-A and other growth factors (M5). Whereas Jagged1 levels were comparable, in the presence of VEGF-A we detected a band of about 65 kDa compatible with the size of mature Dll4 which was not modulated by E2 (Figures 5A and S6E). The increase of the 65 kDa Dll4 protein band (Figures 5B and S6F) and Dll4 mRNA (Figure 5C) was dependent on VEGF-A concentration, indirectly confirming the identity of the 65 kDa band. In VEGF-A-containing medium we were able to detect the active form of Notch1 using the antibody specific for valine 1774. In the presence of E2, the induction of Notch1 processing was even more pronounced (∼52% increase of Notch1IC) and this effect was blocked by ERs pure antagonist ICI 182.780 (Figure 6A, B). Notch2 and 4 protein levels weren’t affected by E2 in the presence of VEGF-A (data not shown). E2 treatment resulted in a modest but statistically significant increase of Hey2 (1.3-fold), Hes4 (1.2-fold) and Dll4 (1.2-fold) mRNAs compared to control, whereas Hes1 and Hey1 were not significantly affected (Figure 6C). Microscopy analysis of immunofluorescence staining for Notch1 in E2 treated cells under M5 conditions, showed reduced Notch1 on the cell membrane (Figure 7A) and a significant increase in the number of cells with nuclear Notch1IC compared to untreated control (39.0±3.76 vs 9.3±1.22) (Figure 7B). It has been shown that in HUVECs, VEGF-A enhances Notch1 activation [7] and induces Dll4 transcription [34]. Thus, to evaluate if the presence of VEGF-A was sufficient to induce the changes observed under M5 conditions, we added VEGF-A to cells grown under M4 conditions and measured expression levels of Dll4 mRNA and of the active form of Notch1. Our results confirmed that addition of VEGF-A to M4 conditions suffices to increase Dll4 mRNA expression and Notch1 activation (Figures S4A, B and S6I). Taken together, these experiments indicate that E2 enhances Dll4-mediated Notch1 activation downstream of VEGF-A and suggest that estrogen receptors ERα or ERβ play a role in this modulation.

### 17β-Estradiol Modulates Notch-regulated Tube Formation and Vascular Sprouting

**Figure 8:**
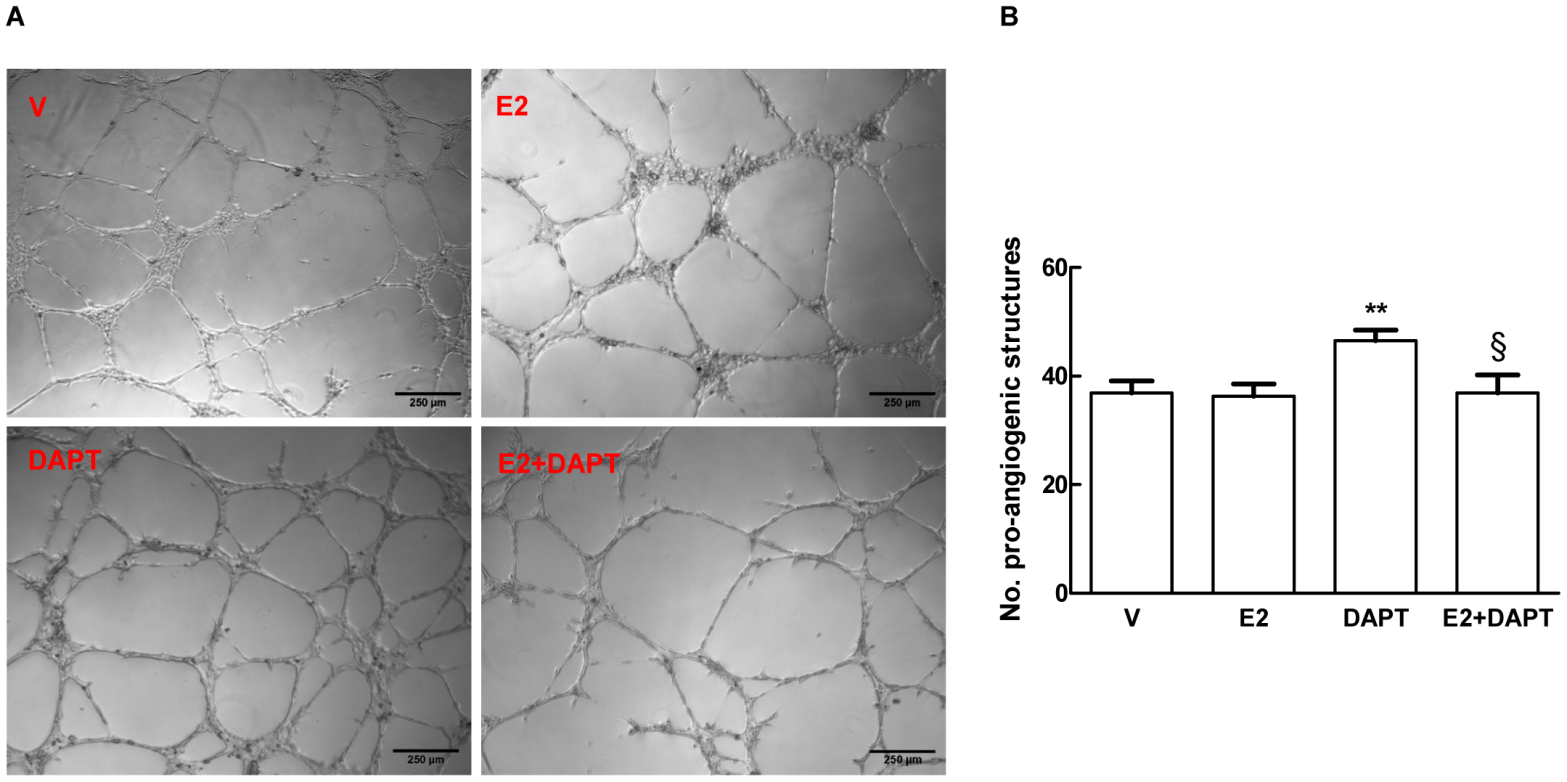
E2 treatment counteracts Notch inhibition- induced endothelial branching *in vitro*. HUVECs were hormone-stripped and treated overnight with 1 nM E2, 5 µM DAPT and a combination of the two. Treatment with DMSO (V) was used as control. The following day 9×10^4^ cells were seeded on 400 µl of Matrigel and treatment continued for additional 8 hours. Number of closed circles was quantified in eight fields after 8 hours of network formation. One representative picture of three different experiments is shown (A) with the respective counts (B). Data are expressed as mean ± SEM. **P<0.01, DAPT significantly different from the control, §P<0.05 E2 plus DAPT significantly different from DAPT.

**Figure 9:**
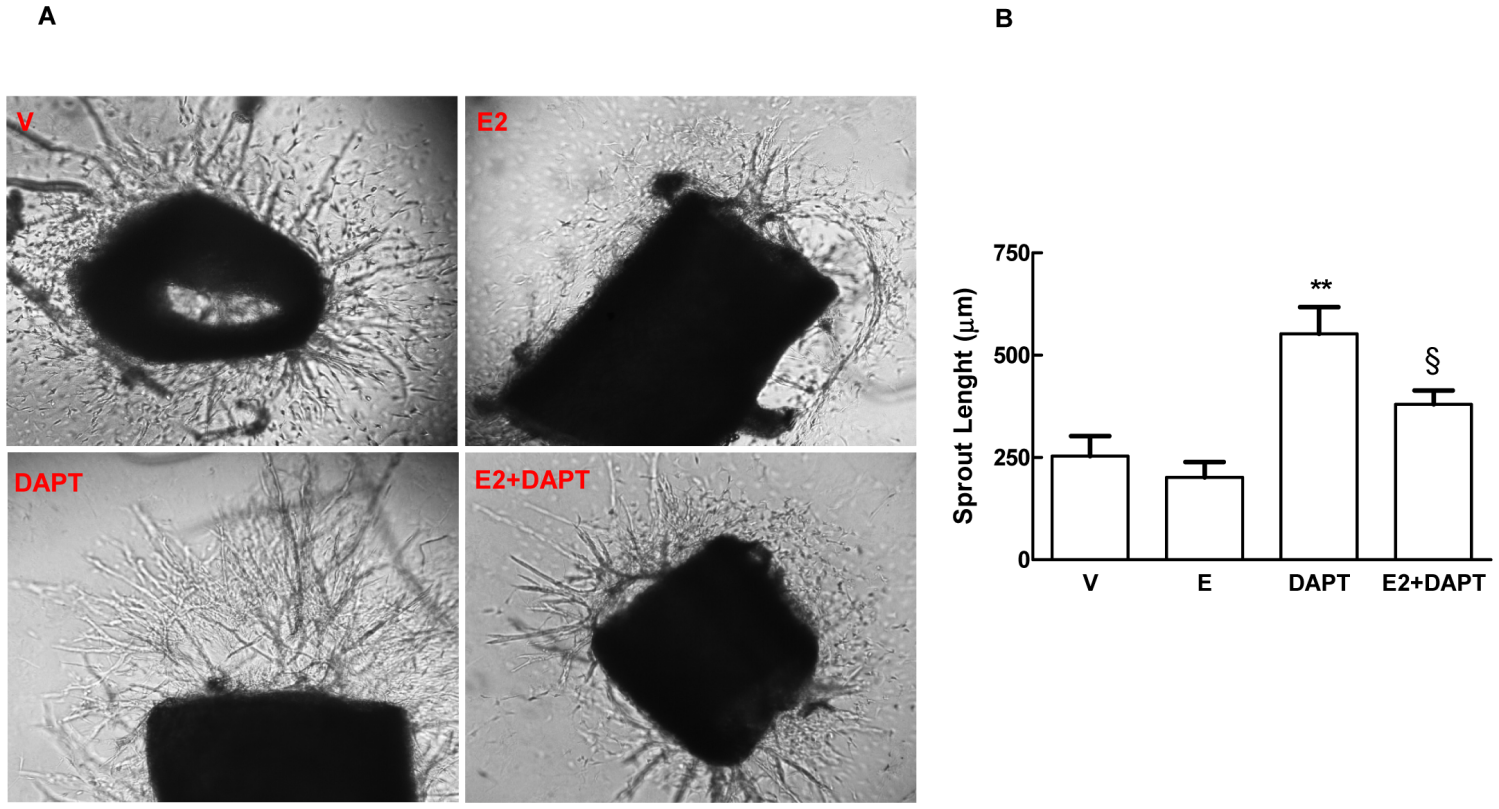
E2 treatment counteracts Notch inhibition- induced vascular sprouting in collagen-based aortic ring explants. Aortic ring explants were embedded in collagen gels and cultured for 5 days in 2.5% csFBS medium containing 30 ng/ml of VEGF-A and treated with 1 nM E2, 5 µM DAPT or 1 nM E2 plus 5 µM DAPT. Treatment with DMSO (V) was used as control. Vascular sprouting was quantified by digital microscopy after 5 days of treatment by measuring the greatest length of sprouts from the body of the aortic ring at three distinct points per ring and on three rings per treatment. One representative picture of three different experiments is shown (A) with the respective sprout lengths (B). Data are expressed as mean ± SEM. **P<0.01, DAPT significantly different from the control, §P<0.05, E2 plus DAPT significantly different from DAPT.

Notch signalling modulates sprouting of new blood vessels by regulating the balance between tip and stalk cells [5]. To determine the possible effects on angiogenesis of the observed E2-dependent VEGF-A/Dll4/Notch1 axis enhancement, we utilized a HUVECs tube formation assay in Matrigel in which the alteration of Notch signalling is reflected in changes in endothelial cells network formation [35]. As indicated by the number of closed intercellular compartments deriving from endothelial cell sprouting (closed rings or pro-angiogenic structures), we did not detect differences in network formation in E2 versus DMSO treated cells (36.2±2.3 vs 36.8±2.2, respectively) suggesting that, under the conditions tested, increased activation of Notch1 by E2 treatment had no effect on endothelial cells sprouting (Figure 8A, B). We found a statistically significant increase in branching of endothelial network in the presence of the Notch activation inhibitor DAPT (46.5±1.9) compared to untreated (36.8±2.2) or E2 (36.2±2.3) treated cells. Co-treatment with DAPT and E2 abolished the effects of DAPT on branching giving a number of closed rings (36.8±3.3) similar to E2 treated or untreated cells (Figure 8A, B). Next we assessed whether E2 had a impact on endothelial cell sprouting using the *ex vivo* aortic ring endothelial cell sprouting assay. In mouse aortic rings explants embedded in collagen and grown in the presence of VEGF-A, 5 days of DAPT treatment caused a dramatic increase in the length of vascular sprouts (552.5±65.1 µm) compared to untreated (232.5±48.7 µm) or E2-treated (201.8±37.5 µm) aortic rings. The effects of DAPT were partially counteracted by co-treatment with E2 (552.5±65.1 µm and 380.3±33.8 µm, respectively) (Figure 9A, B). We didn’t observe differences in the length of sprouts in E2-treated compared to untreated rings (201.8±37.5 µm and 232.5±48.7 µm, respectively) (Figure 9A, B). These effects were still evident after 7 days of treatment (Figure S5A, B). Taken together with the tube formation assay, these data show that E2 modulates sprouting angiogenesis under conditions of reduced Notch1 signalling.

## Discussion

A large number of studies have shown Notch involvement in endothelial cell activation, apoptosis and proliferation. While the effects of various mediators of cell growth or cell activation on Notch pathway in endothelial cells have been well characterized [36] [37], less is known on how estrogens affect Notch in the endothelium.

In this paper, in agreement with previous reports, we show that 17β-estradiol, the most potent naturally occurring estrogen, modulates Notch signalling in HUVECs. We observed augmented levels of active Notch1 and Notch4 and reduction of active Notch2. Among ligands, Dll1 and Dll4 were not affected, whereas Jagged1 protein levels were decreased. We found Hey2, Dll4 and Hes4 mRNAs up-regulated by E2 when Notch signalling was activated by Dll4 but not by Jagged1. Our data indicate that E2 specifically enhances the Dll4-mediated activation of Notch signalling, consistently with previous observations in HUVECs in which overexpression of Dll4 induces Hey2 but not Hey1 or Hes1 mRNAs [32]. We didn’t investigate the underlying mechanism(s) by which E2 specifically affects Notch signalling depending on the particular ligand. It is well known that Notch-ligand interaction can be modulated by the addition of fucose residues to the extracellular EGF-like repeats, which can be further modified by Fringe family β-1,3-N-acetyl-glucosaminyltransferases [38]. In endothelial cells, this glycosylation of Notch leads to enhanced Dll4-Notch signalling and decreased Jagged1-Notch signalling [39]. Noteworthy, Sobrino et al. have shown that 17β-estradiol induces transcription of Radical Fringe [16], suggesting higher levels of glycosylated Notch in E2-treated HUVECs, and a predominance of Dll4-Notch activity. This may partly explain our observation of a preferential enhancement of Dll4 rather than Jagged1-mediated Notch activation by E2. In this study we have analyzed specifically canonical Notch target genes. Since Notch-target genes are notoriously context-dependent [40], [41], more studies are needed to identify which other Notch target genes are modulated in endothelial cells following activation of Notch signalling by E2. Since Hao et al. have shown that Notch1 enhances ER transcriptional activity in breast cancer cells [42], it is also possible that Notch activation by E2 modulates transcription of ER target genes in the endothelium.

We found that addition of ICI 182.780 partially reverses the effects of E2 on processing of Notch1 suggesting that the action of E2 in HUVECs is mediated by one or both estrogen receptors. Our report is in contrast with other findings of Notch inhibition by E2 treatment in ERα positive breast cancer cells [10] and hippocampus [14]. It should be noted that ERα positive breast cancer cell lines and hippocampus express mainly ERα whereas HUVECs express equal levels of ER αand ERβThe two receptors have different and sometimes opposite effects on gene regulation [43] and our results would suggest that ERα and ERβ affect Notch signalling in opposite ways. More studies are needed to confirm that 17β-estradiol activates Notch processing in HUVECs through the estrogen receptor and to identify the mechanism of increased processing. Our data show reduction of transcription of Notch2 but not of Notch1 and 4. E2 mediated up-regulation of Furin mRNA [16] or ADAM17 [44] or ADAM10 [45] could explain enhanced Notch1 and 4 processing. Since Jagged1 is endocytosed following receptor activation [46], [47], the reduced levels of Jagged1 in the presence of E2 are consistent with an augmented processing of Notch1 and 4. Additionally, since VEGF-A increases active Notch1 by Akt-mediated activation of the γ-secretase complex [7], E2-mediated Akt activation [48] could explain our finding of increased Notch1 activation by E2 in the presence of VEGF-A.

Our data confirm and expand previous observations showing activation of Notch signalling by E2 [15], [16] and suggesting that the Notch1 pathway might be implicated in 17β-estradiol regulation of angiogenesis [15]. Under our conditions, we didn’t detect Notch1 and Jagged1 [15] or Notch4 [16] mRNA induction by E2. Whereas these discrepancies could be due to different cell culture conditions, the different methods utilized to study Notch activation (qRT-PCR versus standard RT-PCR [15] or microarrays [16]) could also contribute to explain contrasting results.

**Figure 10:**
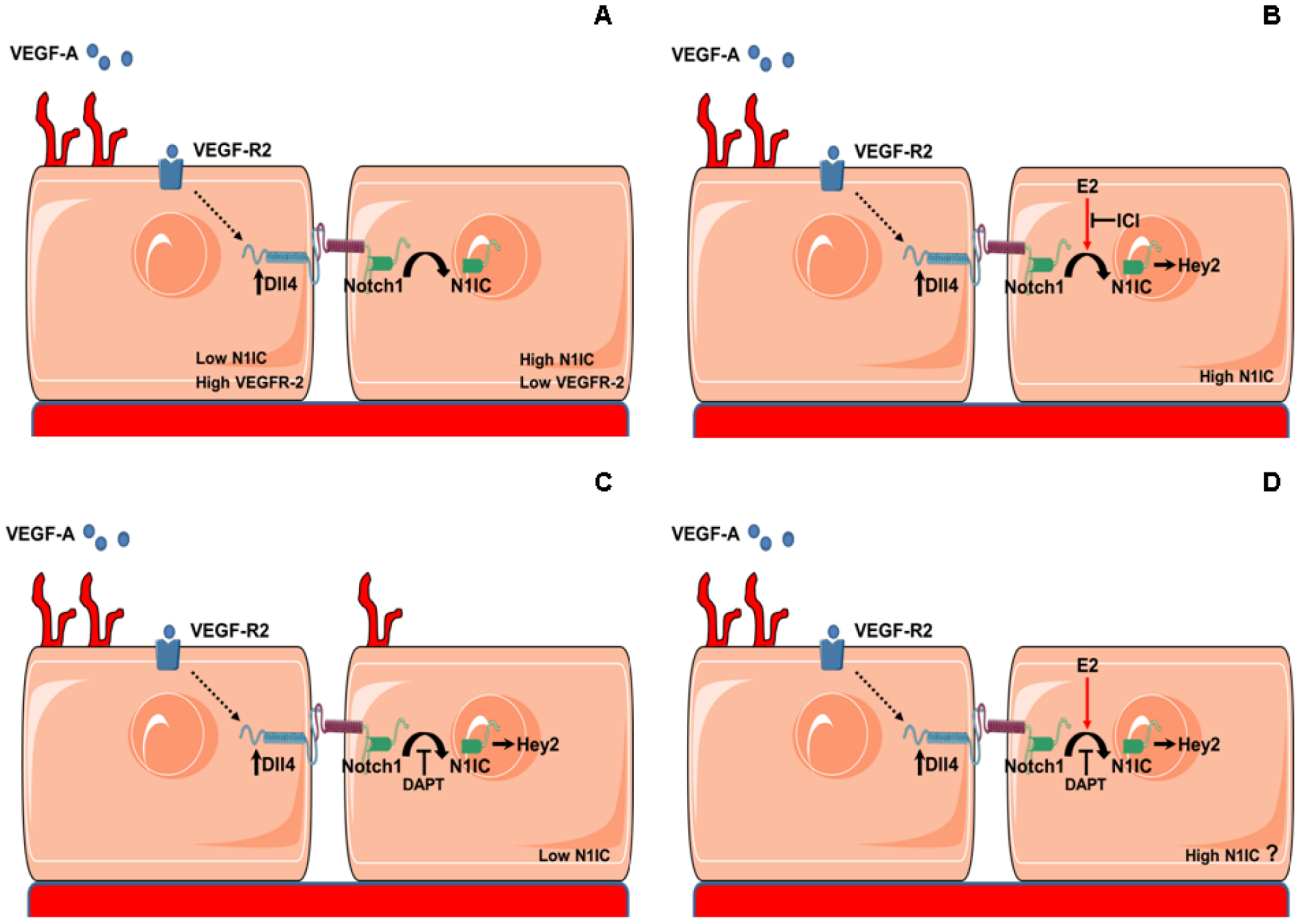
Proposed model for E2-mediated activation of VEGF-A-Dll4-Notch1 signalling and consequences on sprouting angiogenesis in HUVECs. According to a widely accepted model [5] Vascular Endothelial Growth Factor A (VEGF-A), by binding to VEGF Receptor-2 (VEGF-R2), promotes sprouting on endothelial cells and induces expression of Dll4 which, by activating Notch1 in adjacent cells leads to reduction of VEGF-R2 and inhibition of sprouting (A). E2 treatment enhances VEGF-A-mediated increase of the active form of Notch1 (N1IC), effect antagonized by the estrogen receptor antagonist ICI 182.780. In the presence of active Notch1 signalling, a further increase of N1IC induced by E2 does not affect sprouting (B). Notch1 inhibition by treatment with DAPT causes increased sprouting (C). Co-treatment with E2 counteracts DAPT-induced increase in vascular sprouting through still undetermined mechanisms (D).

The enhancement of Dll4-Notch activity by E2 could have important implications in physiological and pathological conditions. Dll4 is predominantly expressed in the vasculature and interacts with Notch1, 2 and 4 receptors [32]. Sprouting of endothelial cells driven by VEGF-A-mediated activation of VEGF-R2 is one of the first steps of neo-angiogenesis. VEGF-R2 activation also induces Dll4. Dll4-activated Notch signalling in the adjacent cells leads to decreased expression levels of VEGF-R2 and to a limitation of sprouting. This interplay between Dll4-Notch and VEGF-A is a major modulator of angiogenesis [4], [5]. Since Notch dampens the VEGF-A response, Notch activation has been associated to a diminished angiogenesis [32]. Recent studies have actually shown that inhibition of Notch by antibodies blocking Dll4 leads to enhanced sprouting but disorganized, functionally defective angiogenesis in tumors and ischemic tissues, indicating instead that a fine-tuned modulation of Notch activity is necessary for a productive angiogenesis [49], [50]. Consistently, in a mouse model of ischemic limb, VEGF-A promotes angiogenesis by increasing γ-secretase activity and Notch1 activation [7]. The pro-angiogenic activity of estradiol has been shown in different contexts [51]. We found that treatment with the Notch activation inhibitor DAPT strongly enhanced HUVECs tubes formation in 3D culture in Matrigel, in agreement with previous work showing excessive network branching in endothelial cells treated with Dll4 siRNA in the same assay [35]. The enhanced branching caused by DAPT was inhibited by co-treatment with E2. Similar results were obtained in an *ex-vivo* assay utilizing mouse aortic rings embedded in collagen in which DAPT induced a dramatic increase in vascular sprouting which was inhibited by co-treatment with E2. In both assays E2-only treatment didn’t have an effect on endothelial cells sprouting. Our data suggest a model whereby in the presence of basal levels of Notch1 activity, a further increase of active Notch1 induced by E2 is not reflected in changes in endothelial sprouting. DAPT, by inhibiting Notch1, increases endothelial cell response to VEGF-A and, consequently, vascular sprouting. In absence of Notch reactivation, this would lead to disorganized, defective angiogenesis. E2 co-treatment could restore basal levels of network branching by counteracting the inhibitory effect of DAPT on Notch1 activation which would in turn lead to reduced response to VEGF-A (Figure 10). In HUVECs in 2D culture, E2 co-treatment didn’t re-establish basal levels of the active form of Notch1 (Figure 6A, B), but it cannot be excluded that in the different growth conditions of 3D culture, E2 could be able to antagonize DAPT-induced Notch1 inhibition. Further studies are therefore needed to verify our hypothesis and/or to identify the alternative molecular mechanisms by which E2 counteracts the DAPT-induced vascular sprouting enhancement.

Our observations may have important translational implications. It is widely held that reduced levels of circulating estrogens are the cause of the dramatic increase of incidence of coronary heart disease in women after menopause. It is thought that estrogen may play an important role in the development of the coronary capillary network and in the regulation of physiological angiogenesis, which would exert a protective effect on coronary blood flow [52]. Estrogens could also play a role in this setting by promoting proliferation of endothelial progenitors involved in the re-endothelization of damaged tissue [53]. Our data indicates that E2 could be a modulator of sprouting angiogenesis specifically under physiological or pathological conditions associated to decreased Dll4-Notch1 signalling. Since increased levels of tumor necrosis factor-α (TNF-α) during inflammation, promote endothelial cells sprouting through Jagged1-mediated inhibition of Dll4-Notch1 signalling [54], it would be interesting to investigate whether estrogens play a role in the modulation of sprouting angiogenesis under inflammatory conditions.

A recent study [55] has confirmed previous findings suggesting that hormone replacement therapy (HRT) administered before the onset of menopause has a protective action on the cardiovascular system [56]. The small increase in the risk of breast cancer associated with HRT [57], [58] is nevertheless a concern for clinicians and patients and limits the use and the potential benefits of HRT. Since our findings suggest an activation of Notch signalling by E2 through a specific ER, selective estrogen receptor modulators (SERMs) designed to target specifically ER-Notch cross-talks in endothelial cells could represent new approaches to promote cardiovascular protection. On a different note, data here reported on the cross-talks between Notch and ER in endothelial cells suggest that it would be of interest to determine the possible effects on angiogenesis of currently used drugs for cancer therapy (aromatase inhibitors and/or ER antagonists) or under clinical investigation (γ-secretase complex inhibitors [59]–[62]), which are targeting either one of these pathways.

## Supporting Information

Figure S1 Positive control to verify immunoreactivity of cleaved Notch1 (Val1744) antibody. HUVECs were treated with 1 nM E2 or DMSO (V) for 24 hours under M4 experimental conditions (2% FBS overnight followed by 20% csFBS). Cell lysates were electrophoresed and immunoblotted with cleaved Notch1 (Val1744) antibody to detect the active form of Notch1 (Notch1IC). 5 mM EDTA treated cells were used as positive control. β-actin antibody was used to ensure equal loading.
(TIF)

Figure S2 Effect of 17β-estradiol treatment on cellular Notch1 localization in HUVECs. (A) Representative microscopy images of HUVECs immunolabelled with Notch1 (C-20) antibody, then treated with 488-conjugated goat anti-rabbit IgG secondary antibody. DAPI staining was used to visualize cell nuclei. Before immunofluorescence staining, cells were treated with 1 nM E2 or DMSO (V) for 24 hours under M4 experimental conditions (2% FBS overnight followed by 20% csFBS). (B) Percentage of cells showing high nuclear staining for Notch1. Cells were collected using an Orca-05G2 at full-frame, without binning. Cells were then scored and counted in 50 fields using the Scan^R Analysis software. Results are expressed as mean ± SEM of three independent experiments. *P<0.05, significantly different from the control.
(TIF)

Figure S3 Effect of 17β-estradiol treatment on EDTA-induced Notch1 activation in HUVECs. (A) HUVECs under M4 experimental conditions (2% FBS overnight followed by 20% csFBS) were treated with 5 mM EDTA for 20 minutes before lysis. Cell lysates were electrophoresed and immunoblotted with Notch1 (C-20) antibody to detect the transmembrane form (Notch1TM) and with cleaved Notch1 (Val1744) antibody to detect the active form of Notch1 (Notch1IC). β-actin antibody was used to ensure equal loading. Densitometric analysis of Western blot assay is shown in Figure S6G. (B) HUVECs under M4 experimental conditions (2% FBS overnight followed by 20% csFBS) were treated with 5 mM EDTA for 20 minutes followed by 4 hours in standard medium. Total RNA was extracted and qRT-PCR analysis of Hes1, Hey1 and Hey2 genes expression was performed. Relative changes in mRNA expression levels were calculated according to the 2^−ΔΔCt^ method using RPL13A as reference gene. Results are expressed as mean ± SEM of three independent experiments, each performed in triplicate. *** P<0.001, significantly different from the control. (C) HUVECs under M4 experimental conditions (2% FBS overnight followed by 20% csFBS) were treated with 1 nM E2 or DMSO (V) for 24 hours and, before lysis, with 5 mM EDTA for 20 minutes. Cell lysates were electrophoresed and immunoblotted with Notch1 (C-20) antibody to detect the transmembrane form (Notch1TM) and with cleaved Notch1 (Val1744) antibody to detect the active form of Notch1 (Notch1IC). β-actin antibody was used to ensure equal loading. Densitometric analysis of Western blot assay is shown in Figure S6H. (D) HUVECs under M4 experimental conditions (2% FBS overnight followed by 20% csFBS) were treated with 1 nM E2 or DMSO (V) for 24 hours and, before lysis, for 20 minutes with 5 mM EDTA followed by 4 hours in standard medium. Total RNA was extracted and qRT-PCR analysis of Hes1, Hey1 and Hey2 genes expression was performed. Relative changes in mRNA expression levels were calculated according to the 2^−ΔΔCt^ method using RPL13A as reference gene. Results are expressed as mean ± SEM of three independent experiments, each performed in triplicate. *** P<0.001, significantly different from the control.
(TIF)

Figure S4 Control for Figure 5. (A) HUVECs were exposed to different VEGF-A concentrations (20 ng/ml and 50 ng/ml) for 24 hours under M4 experimental conditions (2% FBS overnight followed by 20% csFBS). Cell lysates were electrophoresed and immunoblotted with Dll4 and cleaved Notch1 (Val1744) antibodies. β-actin antibody was used to ensure equal loading. Densitometric analysis of Western blot assay is shown in Figure S6I. (B) HUVECs were treated with VEGF-A (20 ng/ml and 50 ng/ml) for 24 hours under M4 experimental conditions (2% FCS overnight followed by 20% csFCS). Total RNA was extracted and qRT-PCR analysis of Dll4 gene expression was performed. Relative changes in mRNA expression levels were calculated according to the 2^−ΔΔCt^ method using RPL13A as reference gene. Results are expressed as mean ± SEM of three independent experiments, each performed in triplicate. *** P<0.001, significantly different from the control.
(TIF)

Figure S5 E2 treatment counteracts Notch inhibition- induced vascular sprouting in collagen-based aortic ring explants. Aortic ring explants were embedded in collagen gels and cultured for 7 days in 2.5% csFBS medium containing 30 ng/ml of VEGF-A and treated with 1 nM E2, 5 µM DAPT or 1 nM E2 plus 5 µM DAPT. Treatment with DMSO (V) was used as control. Vascular sprouting was quantified by digital microscopy after 7 days of treatment by measuring the greatest length of sprouts from the body of the aortic ring at three distinct points per ring and on three rings per treatment. One representative picture of three different experiments is shown (A) with the respective sprout lengths (B). Data are expressed as mean ± SEM. ***P<0.001, DAPT significantly different from the control, §P<0.05, E2 plus DAPT significantly different from DAPT.
(TIF)

Figure S6 Densitometric and statistical analysis of Western blot assays showed in Figure 2C (A), Figure 3A (B), Figure 3C (C), Figure 4B (D), Figure 5A (E), Figure 5B (F), Figure S3A (G) Figure S3C (H) and Figure S4 (I). Graphs show protein levels after indicated treatment normalized to vehicle levels after adjusting for β-actin loading. Results are expressed as mean ± SEM of three independent experiments.*P<0.05, **P<0.01, ***P<0.001 significantly different from the control.
(TIF)
