# Supplementary figures and images for: 17β-Estradiol Enhances Signalling Mediated by VEGF-A-Delta-Like Ligand 4-Notch1 Axis in Human Endothelial Cells
Source: PLoS One. 2013 Aug 13;8(8):e71440. doi: 10.1371/journal.pone.0071440 (PMC3742772; doi:10.1371/journal.pone.0071440)

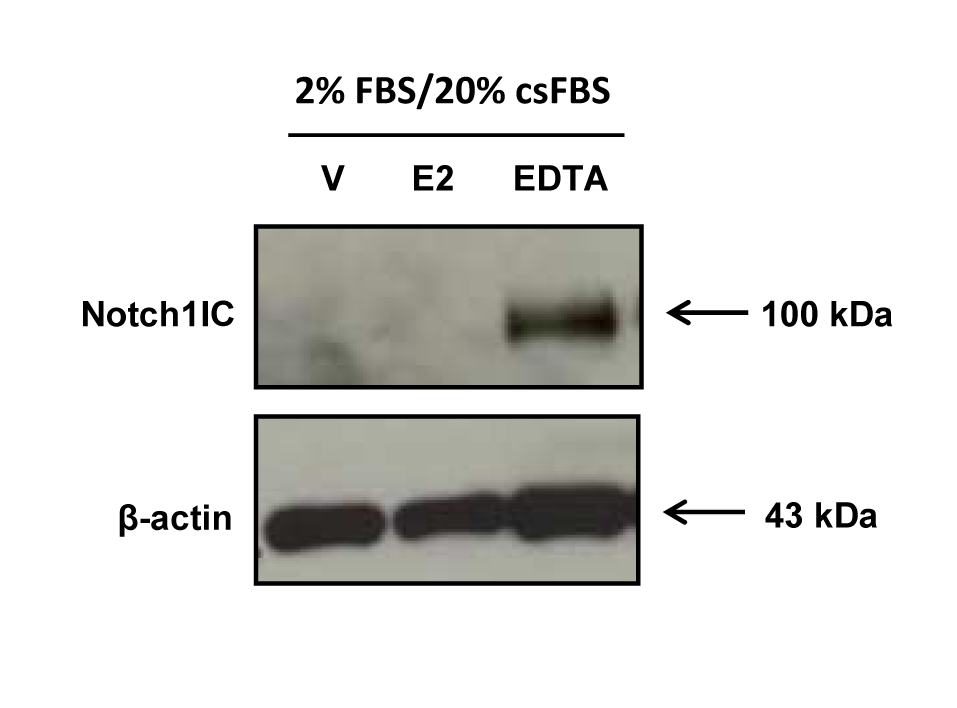

Supplement: Figure S1 — Positive control to verify immunoreactivity of cleaved Notch1 (Val1744) antibody. HUVECs were treated with 1 nM E2 or DMSO (V) for 24 hours under M4 experimental conditions (2% FBS overnight followed by 20% csFBS). Cell lysates were electrophoresed and immunoblotted with cleaved Notch1 (Val1744) antibody to detect the active form of Notch1 (Notch1IC). 5 mM EDTA treated cells were used as positive control. β-actin antibody was used to ensure equal loading. (TIF) [file pone.0071440.s001.tif]

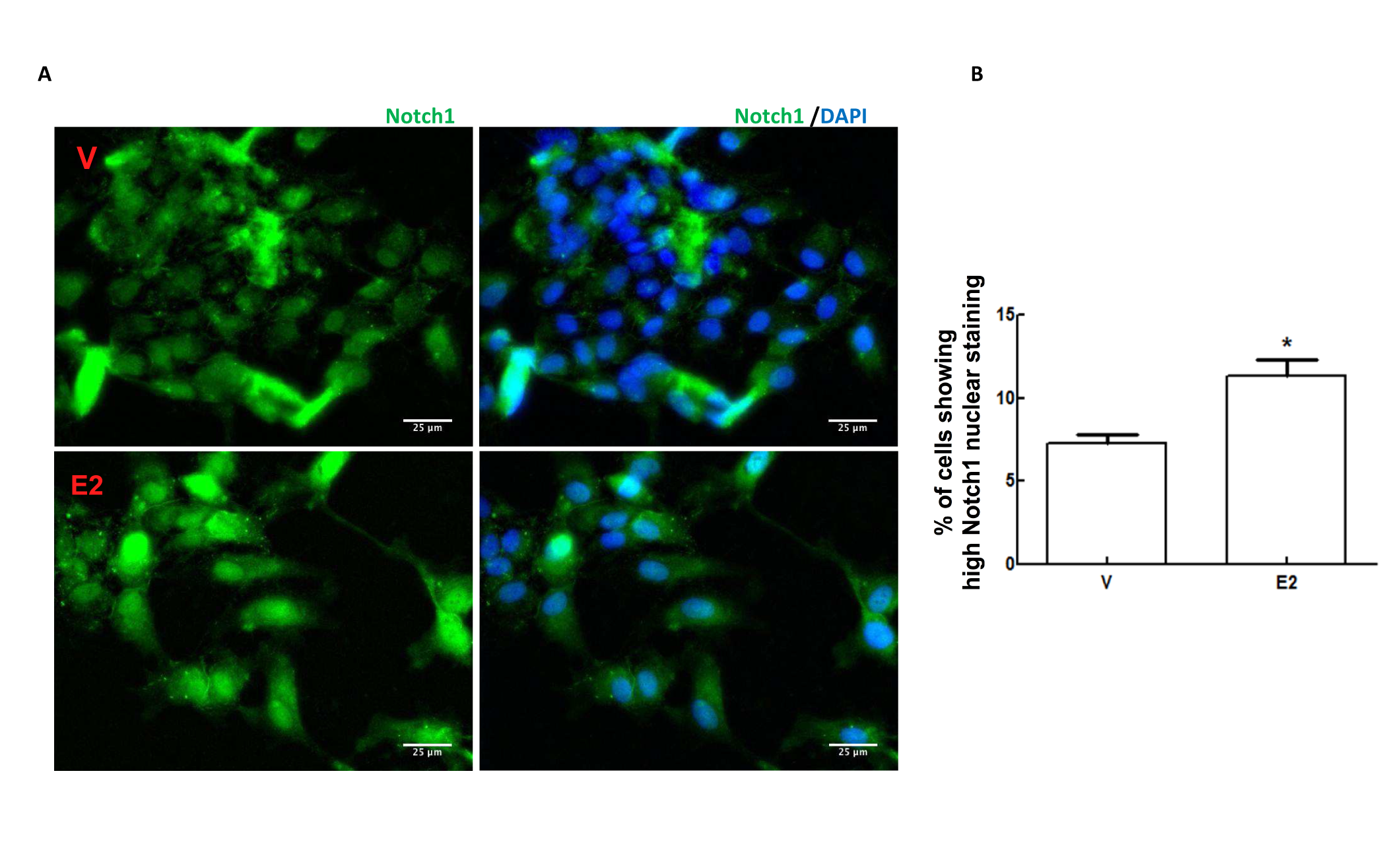

Supplement: Figure S2 — Effect of 17β-estradiol treatment on cellular Notch1 localization in HUVECs. (A) Representative microscopy images of HUVECs immunolabelled with Notch1 (C-20) antibody, then treated with 488-conjugated goat anti-rabbit IgG secondary antibody. DAPI staining was used to visualize cell nuclei. Before immunofluorescence staining, cells were treated with 1 nM E2 or DMSO (V) for 24 hours under M4 experimental conditions (2% FBS overnight followed by 20% csFBS). (B) Percentage of cells showing high nuclear staining for Notch1. Cells were collected using an Orca-05G2 at full-frame, without binning. Cells were then scored and counted in 50 fields using the Scan^R Analysis software. Results are expressed as mean ± SEM of three independent experiments. *P<0.05, significantly different from the control. (TIF) [file pone.0071440.s002.tif]

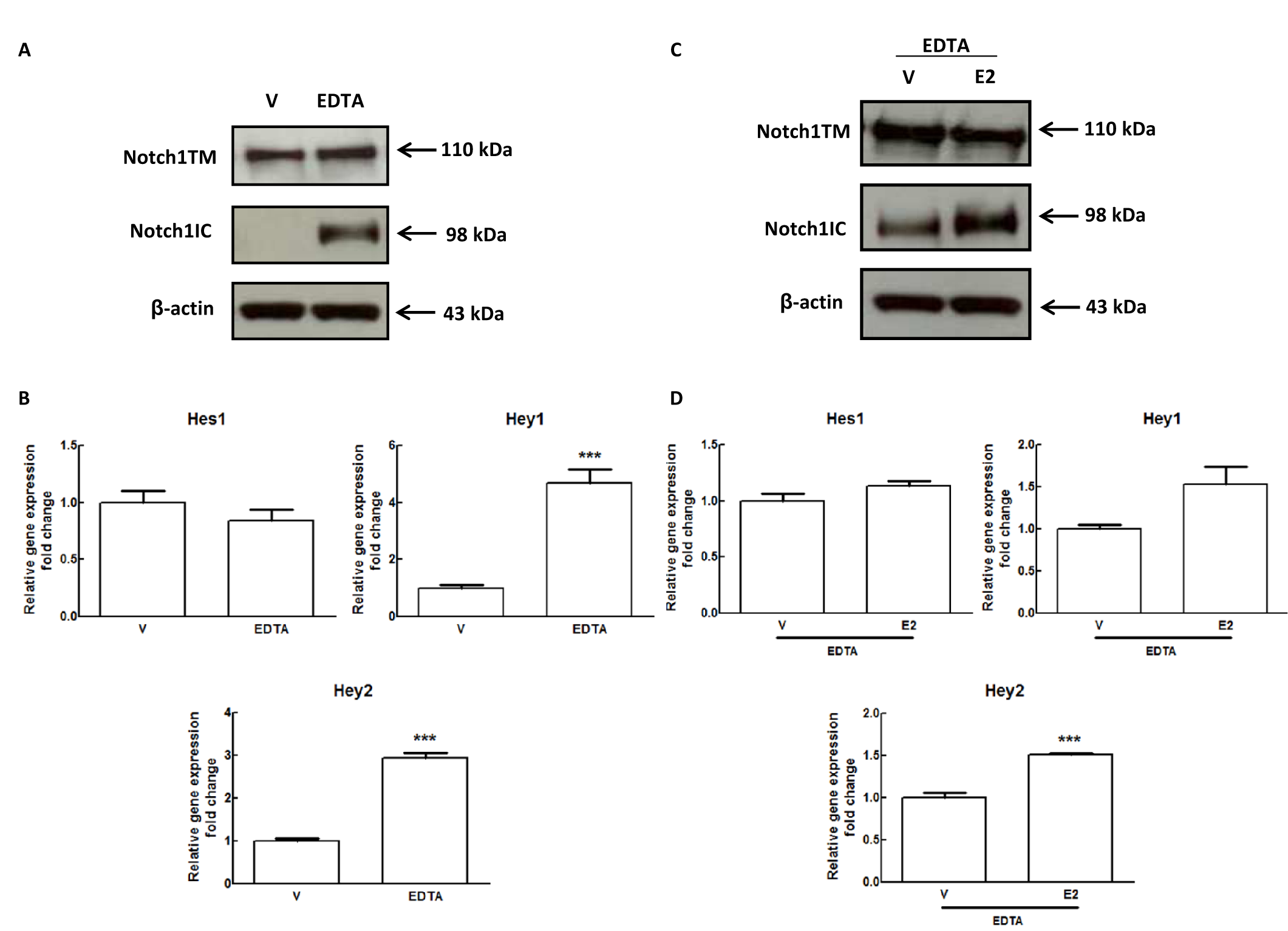

Supplement: Figure S3 — Effect of 17β-estradiol treatment on EDTA-induced Notch1 activation in HUVECs. (A) HUVECs under M4 experimental conditions (2% FBS overnight followed by 20% csFBS) were treated with 5 mM EDTA for 20 minutes before lysis. Cell lysates were electrophoresed and immunoblotted with Notch1 (C-20) antibody to detect the transmembrane form (Notch1TM) and with cleaved Notch1 (Val1744) antibody to detect the active form of Notch1 (Notch1IC). β-actin antibody was used to ensure equal loading. Densitometric analysis of Western blot assay is shown in Figure S6G. (B) HUVECs under M4 experimental conditions (2% FBS overnight followed by 20% csFBS) were treated with 5 mM EDTA for 20 minutes followed by 4 hours in standard medium. Total RNA was extracted and qRT-PCR analysis of Hes1, Hey1 and Hey2 genes expression was performed. Relative changes in mRNA expression levels were calculated according to the 2−ΔΔCt method using RPL13A as reference gene. Results are expressed as mean ± SEM of three independent experiments, each performed in triplicate. *** P<0.001, significantly different from the control. (C) HUVECs under M4 experimental conditions (2% FBS overnight followed by 20% csFBS) were treated with 1 nM E2 or DMSO (V) for 24 hours and, before lysis, with 5 mM EDTA for 20 minutes. Cell lysates were electrophoresed and immunoblotted with Notch1 (C-20) antibody to detect the transmembrane form (Notch1TM) and with cleaved Notch1 (Val1744) antibody to detect the active form of Notch1 (Notch1IC). β-actin antibody was used to ensure equal loading. Densitometric analysis of Western blot assay is shown in Figure S6H. (D) HUVECs under M4 experimental conditions (2% FBS overnight followed by 20% csFBS) were treated with 1 nM E2 or DMSO (V) for 24 hours and, before lysis, for 20 minutes with 5 mM EDTA followed by 4 hours in standard medium. Total RNA was extracted and qRT-PCR analysis of Hes1, Hey1 and Hey2 genes expression was performed. Relative changes in mRNA expression levels were calcu [file pone.0071440.s003.tif]

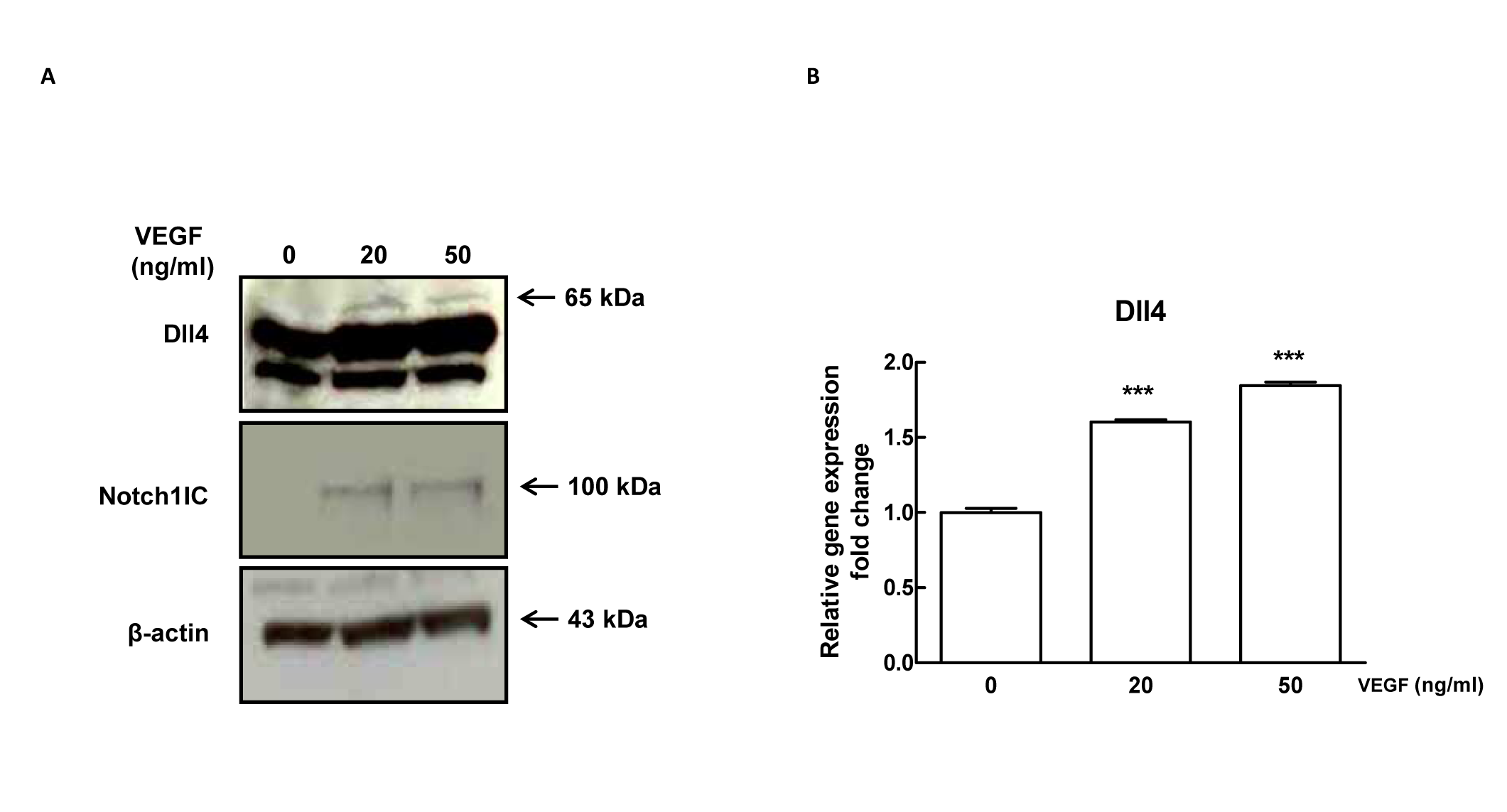

Supplement: Figure S4 — Control for Figure 5. (A) HUVECs were exposed to different VEGF-A concentrations (20 ng/ml and 50 ng/ml) for 24 hours under M4 experimental conditions (2% FBS overnight followed by 20% csFBS). Cell lysates were electrophoresed and immunoblotted with Dll4 and cleaved Notch1 (Val1744) antibodies. β-actin antibody was used to ensure equal loading. Densitometric analysis of Western blot assay is shown in Figure S6I. (B) HUVECs were treated with VEGF-A (20 ng/ml and 50 ng/ml) for 24 hours under M4 experimental conditions (2% FCS overnight followed by 20% csFCS). Total RNA was extracted and qRT-PCR analysis of Dll4 gene expression was performed. Relative changes in mRNA expression levels were calculated according to the 2−ΔΔCt method using RPL13A as reference gene. Results are expressed as mean ± SEM of three independent experiments, each performed in triplicate. *** P<0.001, significantly different from the control. (TIF) [file pone.0071440.s004.tif]

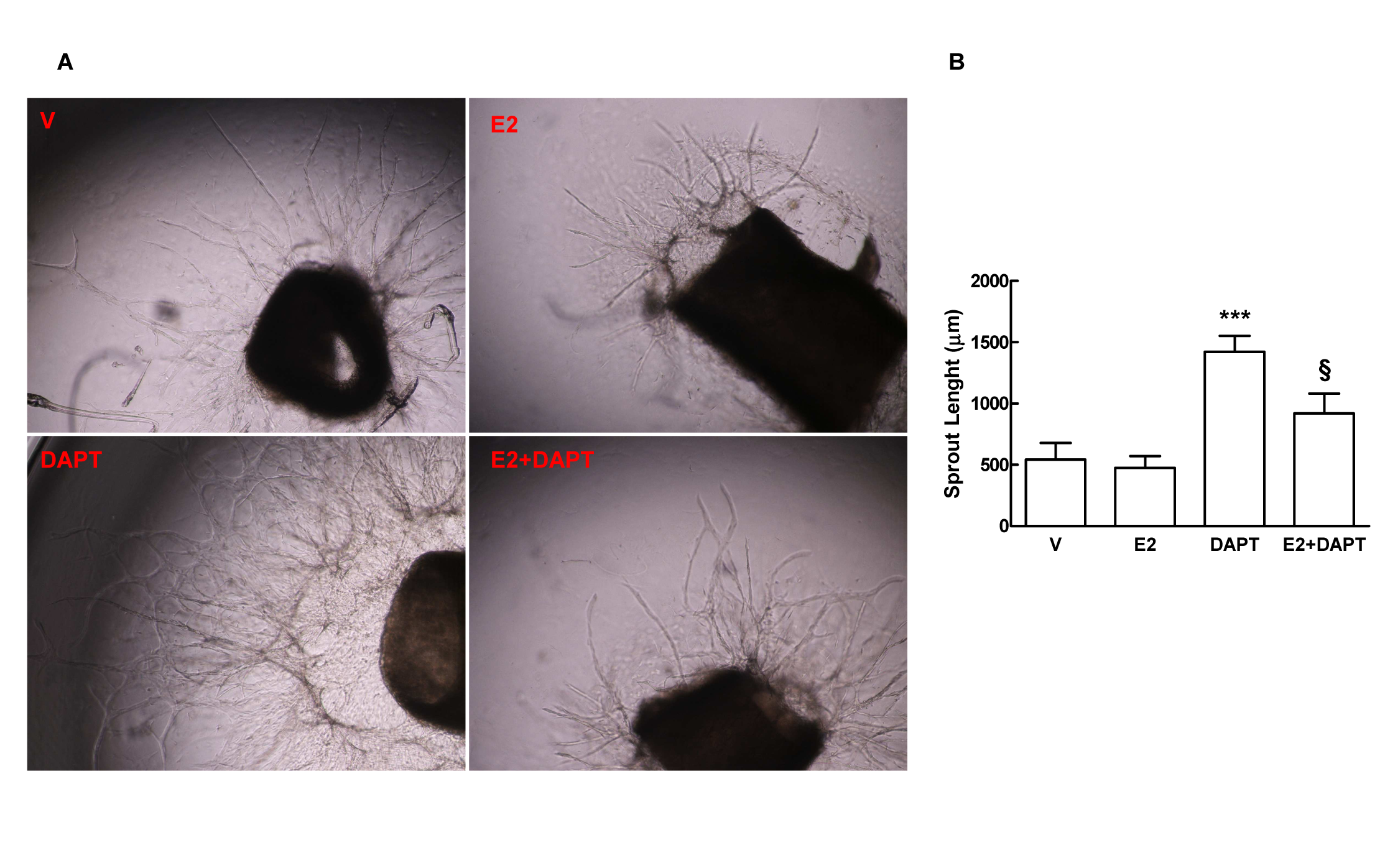

Supplement: Figure S5 — E2 treatment counteracts Notch inhibition- induced vascular sprouting in collagen-based aortic ring explants. Aortic ring explants were embedded in collagen gels and cultured for 7 days in 2.5% csFBS medium containing 30 ng/ml of VEGF-A and treated with 1 nM E2, 5 µM DAPT or 1 nM E2 plus 5 µM DAPT. Treatment with DMSO (V) was used as control. Vascular sprouting was quantified by digital microscopy after 7 days of treatment by measuring the greatest length of sprouts from the body of the aortic ring at three distinct points per ring and on three rings per treatment. One representative picture of three different experiments is shown (A) with the respective sprout lengths (B). Data are expressed as mean ± SEM. ***P<0.001, DAPT significantly different from the control, §P<0.05, E2 plus DAPT significantly different from DAPT. (TIF) [file pone.0071440.s005.tif]

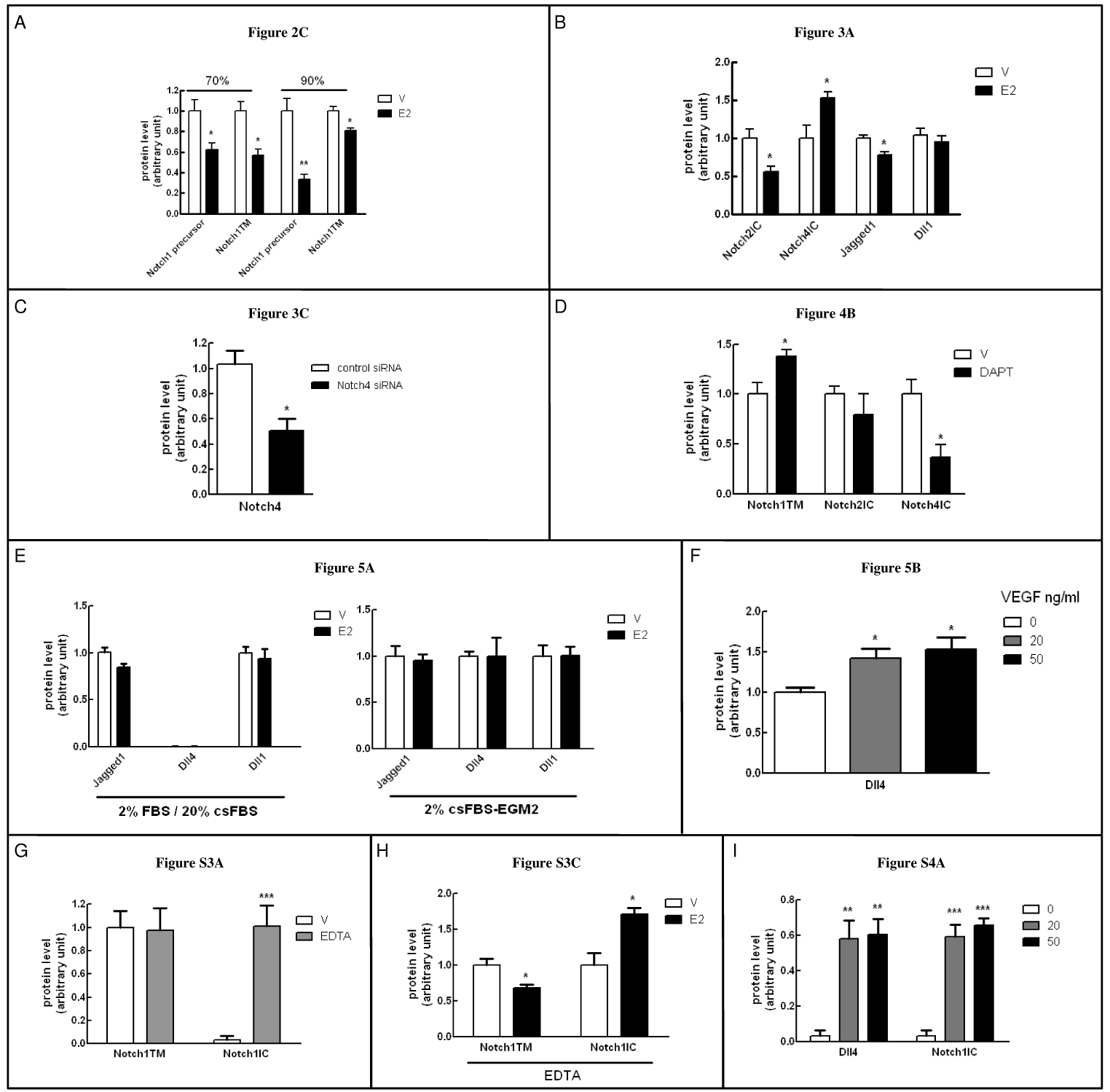

Supplement: Figure S6 — Densitometric and statistical analysis of Western blot assays showed in Figure 2C (A), Figure 3A (B), Figure 3C (C), Figure 4B (D), Figure 5A (E), Figure 5B (F), Figure S3A (G) Figure S3C (H) and Figure S4 (I). Graphs show protein levels after indicated treatment normalized to vehicle levels after adjusting for β-actin loading. Results are expressed as mean ± SEM of three independent experiments.*P<0.05, **P<0.01, ***P<0.001 significantly different from the control. (TIF) [file pone.0071440.s006.tif]
